# pH-Triggered Assembly of Endomembrane Multicompartments in Synthetic Cells

**DOI:** 10.1021/acssynbio.1c00472

**Published:** 2021-12-10

**Authors:** Félix Lussier, Martin Schröter, Nicolas J. Diercks, Kevin Jahnke, Cornelia Weber, Christoph Frey, Ilia Platzman, Joachim P. Spatz

**Affiliations:** † Department of Cellular Biophysics, Max Planck Institute for Medical Research, Jahnstraße 29, D-69120 Heidelberg, Germany; ‡ Institute for Molecular Systems Engineering (IMSE), Heidelberg University, Im Neuenheimer Feld 225, D-69120 Heidelberg, Germany; § Biophysical Engineering Group, Max Planck Institute for Medical Research, Jahnstraße 29, D-69120 Heidelberg, Germany; ∥ Department of Physics and Astronomy, Heidelberg University, D-69120 Heidelberg, Germany; ⊥ Max Planck School Matter to Life, Jahnstraße 29, D-69120 Heidelberg, Germany

**Keywords:** synthetic biology, self-assembly, giant unilamellar vesicle, multicompartments, droplet-based microfluidics, water-in-oil emulsion

## Abstract

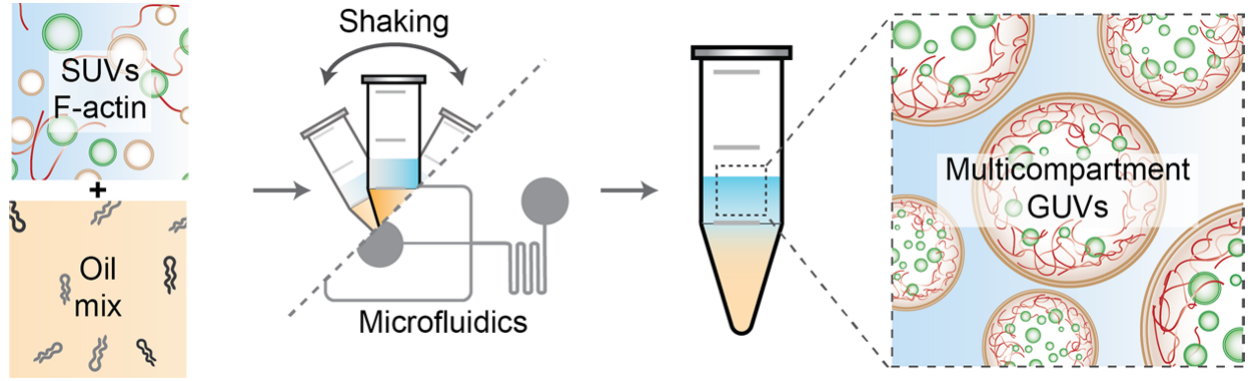

By using electrostatic interactions as driving force to assemble vesicles, the droplet-stabilized method was recently applied to reconstitute and encapsulate proteins, or compartments, inside giant unilamellar vesicles (GUVs) to act as minimal synthetic cells. However, the droplet-stabilized approach exhibits low production efficiency associated with the troublesome release of the GUVs from the stabilized droplets, corresponding to a major hurdle for the droplet-stabilized approach. Herein, we report the use of pH as a potential trigger to self-assemble droplet-stabilized GUVs (dsGUVs) by either bulk or droplet-based microfluidics. Moreover, pH enables the generation of compartmentalized GUVs with flexibility and robustness. By co-encapsulating pH-sensitive small unilamellar vesicles (SUVs), negatively charged SUVs, and/or proteins, we show that acidification of the droplets efficiently produces dsGUVs while sequestrating the co-encapsulated material. Most importantly, the pH-mediated assembly of dsGUVs significantly improves the production efficiency of free-standing GUVs (i.e., released from the stabilizing-droplets) compared to its previous implementation.

## Introduction

Achieving the construction of a living system from nonliving building blocks would tremendously impact various aspects of cellular biology, ranging from revolutionizing our understanding of the origin of life^1^ to the production of man-made artificial cells to fight cancer.^2^ Toward these aims, synthetic biology dissects, isolates, and reconstructs cellular processes through the assembly of well-characterized molecular building blocks. This holistic vision is thus promoting and improving our current understanding of individual cellular functions, but also fostering our ability to investigate their collective and emerging properties. Up to now, various living cell components and functions, such as cytoskeleton,^3,4^ metabolism,^3,5−7^ signaling,^8^ protein expression,^9,10^ growth,^11^ and division^12^ have all been individually reconstructed within cell-sized compartments. To perform and sustain most of these functions, eukaryotes rely on a vast and complex endomembrane system which segregate cellular functions into specialized compartments referred to as organelles. The presence of organelles, and hence the concept of compartmentalization, enables the co-existence of chemically distinct reactions in spatially confined reactors while allowing multistep reactions and sustaining chemical gradients. The construction of a compartmentalized system in synthetic eukaryotes is thus key in order to build more sophisticated synthetic cells for both fundamental biological investigations, and as promising novel biomaterial for healthcare applications.^13−15^

In fact, compartments have already been reconstructed within lipid-based vesicles, often referred to as vesosomes, due to their potential in the field of drug delivery by minimizing passive leakage of therapeutic drugs.^16^ Vesosomes assembly was typically achieved through bulk processes, where the lipid composition, polydispersity, throughput, and reproducibility were limited and hardly controlled.^16−18^ Fortunately, most of these limitations may be circumvented through the usage of droplet-based microfluidics to improve precision and manipulation. Water-in-oil-in-water (W/O/W) emulsions produced by microfluidics technologies were recently applied to encapsulate small^11,19,20^ or large lipid compartments^21^ inside giant unilamellar vesicles (GUVs). Yet, the use of W/O/W emulsions to encapsulate biological materials or compartments relies on an a priori limited sets of lipids [i.e., mostly phosphatidylcholine (PC)-based lipids]. In some cases, the use of nonionic surfactants to foster the spontaneous dewetting of the excess oil from the emulsion by minimizing the total interfacial energy^22,23^ and avoid membrane defects^24,25^ is required, which may impair the mechanical properties of the lipid membrane.

As an alternative, water-in-oil (W/O) emulsions stabilized by a mixture of an uncharged polyethylene glycol (PEG)-based fluorosurfactant and a negatively-charged perfluoropolyether (PFPE) carboxylic acid fluorosurfactant (namely Krytox) were reported to enable the genesis of vesicles, referred to as droplet-stabilized GUVs (dsGUVs).^26,27^ In the droplet-stabilized approach, a net negative charge at the W/O droplet interface is generated due to the accumulation of Krytox surfactants, which initiated the selective recruitment and fusion of small unilamellar vesicles (SUVs) at the droplet periphery in the presence of Mg^2+^ ions.^26^ The droplet-stabilized approach offers the possibility of using a highly complex lipid composition and avoiding the use of surfactants for dewetting, thus minimizing the potential membrane defect. When applied to assembling compartmentalized GUVs, the method relies on a preferential electrostatic interaction: by co-encapsulating cationic and negatively charged SUVs in the absence of Mg^2+^ ions in W/O droplets, Göpfrich et al. showed the selective recruitment and fusion of the cationic SUVs at the droplet periphery due to the negatively charged droplet interface.^26^ The resulting dsGUVs thus sequestered the negative SUVs, which remained within the vesicle lumen. Albeit promising, the need of cationic lipids (i.e., DOTAP) to recreate an endomembrane system in GUVs still lacks flexibility and is intricately unnatural and more cytotoxic.^28,29^ As an alternative to permanent cationic lipids, pH-sensitive lipids bearing chemical functional groups capable of modulating their ionic state as a function of pH could circumvent this problem by reducing their toxicity at physiological pH.^30,31^

Herein, we present the pH-mediated reconstruction of an endomembrane system within GUVs through a W/O emulsion using both bulk and microfluidic approaches. As a proof of concept, the method also enables the co-encapsulation of proteins, herein F-actin, with or without a multicompartment system. These results showcase the potential of the pH-triggered assembly of dsGUVs to reconstruct more than a single cellular component, such as compartments and proteins, an important milestone for the droplet-stabilized method, where the complexity of the synthetic eukaryote can now be incremented. Besides the ability to co-encapsulate different components, the use of pH to trigger the charge-mediated assembly of dsGUVs significantly improves the production efficiency of free-standing GUVs (i.e., released from the stabilizing-droplets) without the needs of Mg^2+^ ions, a major hurdle in the droplet-stabilized method.

## Results and Discussion

### pH-Mediated Assembly of dsGUVs

**Figure 1:**
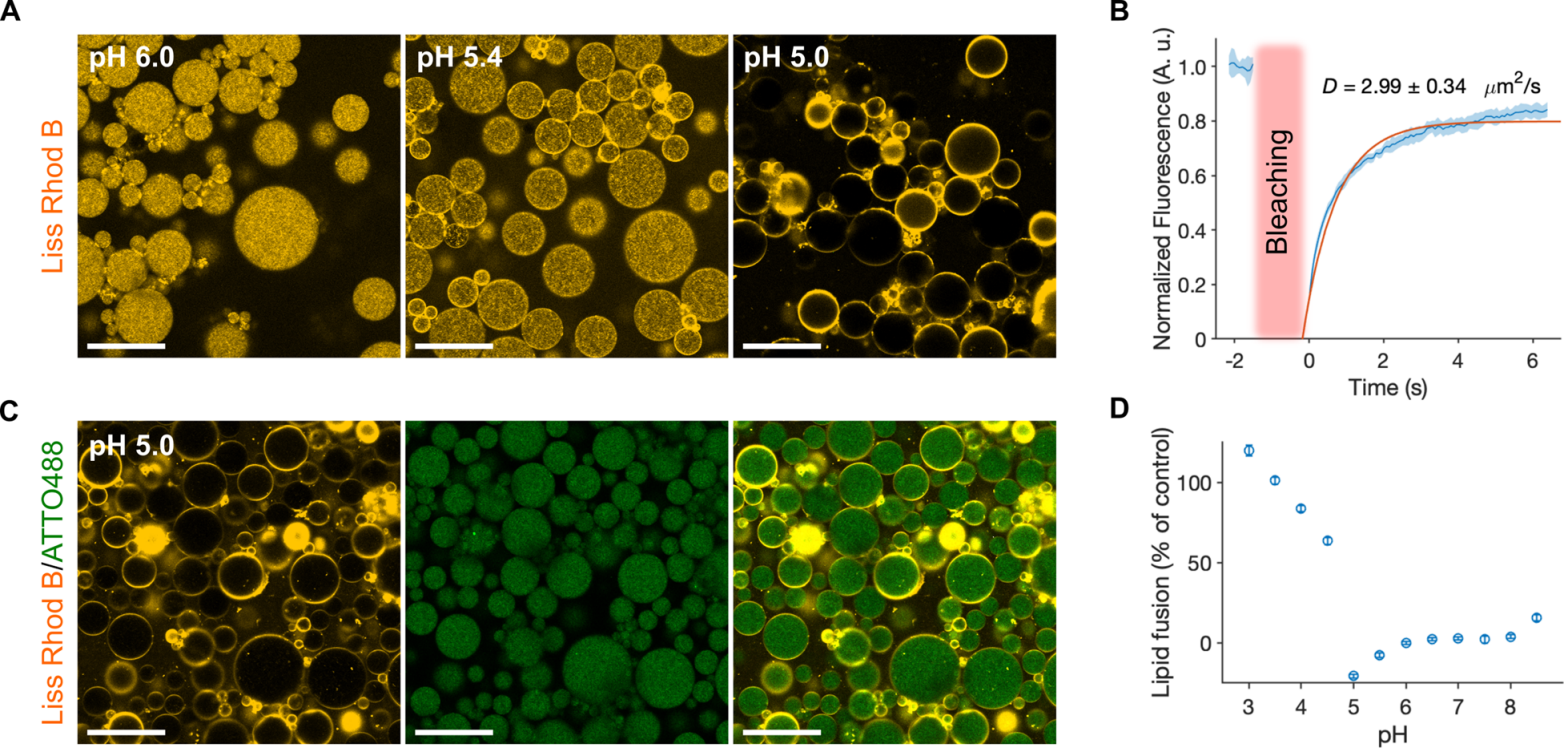
pH-mediated assembly of dsGUVs. (A) Representative confocal images of the encapsulated pH-sensitive SUVs composed of DOBAQ/eggPG/eggPC/Liss Rhod B-labeled DOPE (60/20/19.5/0.5 mol %) within the W/O droplets stabilized by 1.4 wt/wt % PEG-based fluorosurfactant and 10 mM Krytox. Various 50 mM citrate buffers we used to adjust the pH of the water phase. Scale bars: 50 μm. (B) FRAP of dsGUV assembly through a pH trigger. Mean ± S.D. (*n* = 11) are presented. The bleached area is highlighted with the red rectangle. The mean-normalized fluorescence intensity values within the circular bleached area (2.5 μm radius) are plotted as a function of time. The orange line represents an exponential fit (*R*^2^ = 0.9301), which was further used to extract the diffusion coefficient *D* of the lipids in the dsGUVs. The extracted value of *D* was 2.99 ± 0.34 μm^2^/s, similar to the previously reported value for dsGUVs, thus confirming the formation of a supported-lipid bilayer within the W/O droplet. (C) Self-assembly of multicompartment dsGUVs in the presence of 50 mM citrate buffer at pH 5. The pH-sensitive SUVs were co-encapsulated with negatively charged SUVs composed of DOPC/DOPG/ATTO488-labeled DOPE (79.5/20/0.5 mol %). Scale bars: 50 μm. (D) FRET experiment measuring lipid mixing of pH-sensitive SUVs. SUVs composed of DOBAQ/eggPG/eggPC/Liss Rhod B-labeled DOPE/NBD-labeled DOPE (60/20/18/1/1 mol %) were mixed with unlabeled negatively charged SUVs at various pH values. At a pH below 5.0, a significant mixing of the SUVs was observed, depicted by the abrupt rise in fluorescence intensity resulting from the unquenching of the NBD reporter by Liss Rhod B. Mean ± S.D. are presented (*n* = 3).

As a first step, we investigated the potential use of pH to mediate the assembly of dsGUVs in bulk by combining and vortexing the water and oil phases for rapid prototyping of the experimental conditions. Toward this end, SUVs containing the pH-sensitive lipid *N*-(4-carboxybenzyl)-*N*,*N*-dimethyl-2,3-bis(oleoyloxy)propan-1-aminium (DOBAQ) were suspended in citrate buffer at various pH values and encapsulated within W/O droplets stabilized by an oil–surfactant mixture composed of a 2.5 mM PEG-based fluorosurfactant and 10 mM Krytox in HFE-7500 (Figure S1). Imaging by confocal laser scanning microscopy (CLSM) revealed that at pH 6, the fluorescence signal associated to Lissamine rhodamine B (Liss Rhod B)-labeled lipids supplemented to the SUVs was uniformly distributed within the droplets’ lumen (Figure 1A). Upon reduction of the intraluminal pH, we observed an increased recruitment of the pH-sensitive SUVs to the interface of the W/O droplets. A complete recruitment and fusion of the encapsulated SUVs at pH 5 led to the assembly of droplet-stabilized GUVs (Figure 1A). Fluorescence recovery after photobleaching (FRAP) measurements confirmed the successful assembly of a supported lipid bilayer. The measured diffusion coefficient (2.99 ± 0.34 μm^2^/s) of Liss Rhod B-labeled DOPE lipids is similar to the values reported for dsGUVs (Figure 1B).^3,26^

To characterize the fusion behavior of DOBAQ-SUVs as a function of pH at the droplet interface, we first assessed the p*K*_a_ of the DOBAQ lipid in SUVs spectroscopically throughout a 2-(*p*-toluidino)-6-naphthalene sulfonic acid (TNS)-based assay. TNS is a fluorescent reporter whose fluorescence is attenuated in a hydrophilic environment and widely applied to evaluate the p*K*_a_ of lipid-based nanoparticles.^30,32−34^ Due to its intricate negative charge (Figure S2A), TNS is more readily attracted to positively charged lipid membranes. The resulting increase in lipophilicity of the local vicinity unquenches the fluorescence of the TNS probe. Thus, the fluorescence of TNS serves as an indicator of the surface charge of lipid-like vesicles. By varying the pH of the SUVs suspension in the presence of TNS, we evaluated the surface charge of DOBAQ, and hence its p*K*_a_. Through the TNS-assay (see Supporting Information), p*K*_a_ of DOBAQ was estimated to be 4.35 by fitting a sigmoid function (Figure S2B) and by evaluating p*K*_a_ as the point at half-maximum, where 50% of the DOBAQ would be protonated. This value is in good agreement with zeta potential measurement^35^ and fusion assays as a function of pH reported elsewhere.^31^

Up to now, the charge-mediated generation of multicompartment dsGUVs was limited to either the use of cationic SUVs containing DOTAP lipids, which were preferentially recruited at the negatively charged droplet periphery, while entrapping negatively charged SUVs^26^ or via the encapsulation of an excess of negatively charged SUVs in the presence of Mg^2+^ ions.^27^ Here, we investigated the use of pH to trigger the assembly of multicompartment dsGUVs from the bottom-up and avoid the use of cationic lipids permanently (Figure S1; see Table S1 and Supporting Information note 1 for further details on buffer compositions). To this extent, two SUV populations were suspended in various citrate buffers containing no Mg^2+^ ions and co-encapsulated within W/O droplets stabilized by the same oil–surfactant mixture (Figure 1C). Here, one population of SUVs were pH-sensitive (i.e., DOBAQ/eggPG/eggPC/Liss Rhod B-labeled DOPE), while the other SUVs possessed no pH-sensitive motif and were negatively charged at pH values 5 and 7.4 (i.e., DOPC/DOPG/ATTO488-labeled DOPE) (Figure S3). Upon emulsification at pH 5, we observed the selective recruitment and fusion of the pH-sensitive SUVs on the droplet periphery (Figure 1C), while limited to no recruitment was observed at higher pH (Figure S4). Interestingly and importantly, no lipid mixing (i.e., fusion) in between the two SUV populations was detected within the droplets, as the fluorescent signal associated with the pH-sensitive SUVs was solely detected at the droplet periphery at pH 5 (Figure 1C).

Fluorescence resonant energy transfer (FRET) measurements were applied to further understand the preferential fusion of pH-sensitive SUVs to the droplet periphery over fusion with negatively charged SUVs. Toward this end, using the well-established NBD-Liss Rhod B FRET pair, we measured the lipid mixing between the pH-sensitive SUVs and the negatively charged SUVs as a function of pH (Figure 1D). As the pH decreased, a negligible lipid mixing was observed between the SUVs at pH > 5, while an abrupt rise in the fluorescence signal was detected at lower pH. This increase in the fluorescence signal was associated with the unquenching of the NBD-labeled lipid upon mixing. Interestingly, we detected the lowest lipid mixing exactly at pH 5, corroborating the minimal interaction in between SUV population encounter at pH 5 and which then preferentially fuse the pH-sensitive SUVs to the droplet periphery. This low interaction is an improvement compared to the previously reported assembly of multicompartment dsGUVs, employing cationic SUVs in the absence of Mg^2+^ ions.^26^

To compare both systems, we generated the multicompartment dsGUVs by mixing cationic and negatively charged SUVs in the absence of Mg^2+^ at pH 7.4 (Figure S5A).^26^ When produced, cationic SUVs were preferentially recruited at the droplet interface, but also presented a homogeneous fluorescence signal within the droplet’s lumen (Figure S5A). This signal originated from partial lipid mixing between the cationic and negatively charged SUVs, as confirmed by the FRET assay (Figure S5B). Hence, usage of pH-sensitive SUVs rather than permanent cationic lipids to assemble multicompartment dsGUVs corresponds to an improved method due to the minimal lipid mixing in between compartments. Moreover, the pH-sensitive lipids enable the formation of multicompartment free standing GUVs (see section Usage of pH Improves the SUV to GUV Conversion) possessing a net negative charge −30 ± 1 mV (*n* = 3) under physiological conditions as measured by ζ-potential (Figure S3C). This is an important prerequisite for further investigation and usage of GUVs for in vitro studies because cationic lipids are highly potent toward the cellular membrane.^28,29^

### Engineering of Compartmentalized dsGUV Assembly in Bulk and Microfluidics

Cell-sized compartments may be assembled by bulk processes, such as electroformation^36^ or hydration methods,^37,38^ to mimic the physical confinement of cells. When further control and uniformity over the size are required, various droplet-based microfluidics technologies are routinely employed to generate emulsions.^6,7,23,39−41^ Among these methods, the charge-mediated assembly of GUVs using W/O droplets enables a rapid prototyping of various experimental conditions in bulk, while enabling a direct translation to the microfluidic platform. Here, we evaluated if the use of pH to initiate the assembly could impede this translation from bulk to microfluidics.

By co-encapsulating pH-sensitive and negative SUVs within W/O droplets at pH 5, the assembly of compartmentalized dsGUVs in the average size of 12.0 ± 2.7 μm (PDI = 0.098) was achieved by implementation of the double aqueous inlet microfluidic device (Figure S6A–C; video S1), a reduced size, and polydispersity compared to shaking (Figure S7). However, we observed temporal variation in pressures of the inlets due to aggregation between SUVs upon exposition to the citrate buffer prior to their encapsulation in W/O droplets, thus rendering the translation challenging and slightly affecting the homogeneity of the assembled droplets in between experiments (Figures S6D and S7B). We speculate that the clogging was associated with a rapid reduction of the colloidal stability when pH was reduced. In all cases, the pH-sensitive SUVs incorporate the negatively charged lipid DOPG to improve their colloidal stability at pH 7.4 by promoting electrostatic repulsion. With such lipid composition and in the absence of other negatively charged interfaces (i.e., the droplet interface), the SUVs tend to aggregate upon rapid reduction of the environmental pH.

To assemble small cell-sized compartments (≤10 μm), we investigated the use of microfluidic devices possessing a smaller channel width and the translation capability of the methods to various microfluidic modules. Hence, we applied the pH-mediated approach to assemble compartmentalized dsGUVs employing a mechanical splitter, possessing channels of 2 μm as the smallest feature (Figure S8A,B).^35^ With such small channels, we further observed a rapid clogging of the microfluidic channels upon mixing SUVs and citrate buffer, which resulted in poor homogeneity and high polydispersity compared to emulsification by shaking (Figures S7A and S8C,D). Similar results were observed when a 50 mM phosphate buffer composed of KH_2_PO_4_/K_2_HPO_4_ at pH 5 was employed rather than citrate, suggesting that pH may be the dominant effect over buffer composition. Consequently, the use of a low pH buffer resulted in a limited translation of this method to microfluidic platforms possessing small channel geometry and could not be deployed universally.

**Figure 2:**
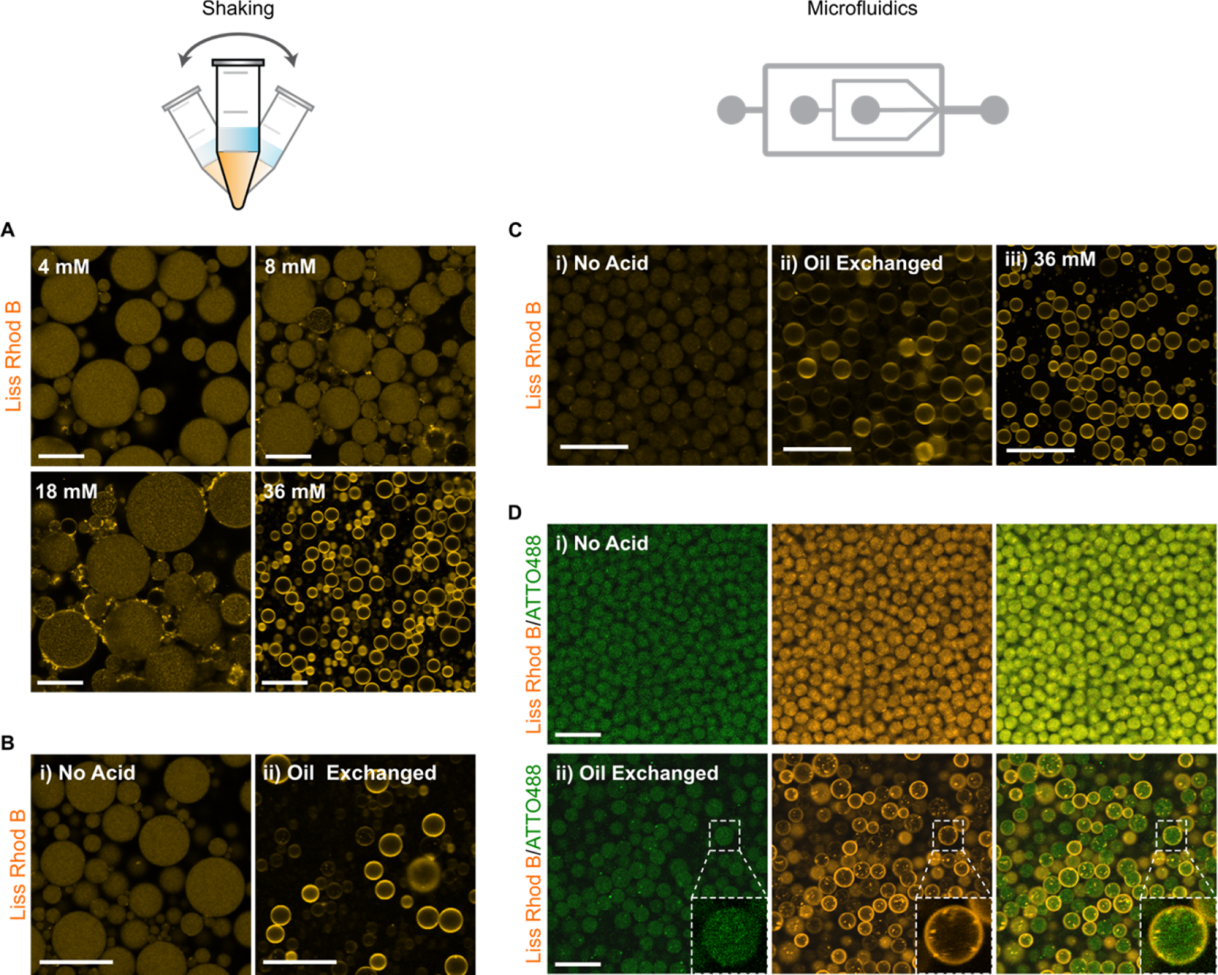
Assembly of dsGUVs by acidification from the oil–surfactant phase via the bulk shaking method or microfluidic technology. (A) Representative confocal fluorescence images of pH-sensitive SUVs [DOBAQ/DOPG/DOPC/Liss Rhod B labeled-DOPE (60/20/19.5/0.5 mol %)] within W/O droplets stabilized by 1.4 wt % PEG-based fluorosurfactant and 10 mM Krytox in HFE-7500 oil that contains various concentrations of acetic acid (4, 8, 18, and 36 mM). Droplets were produced by the bulk shaking method and the aqueous phase consisted of 1.5 mM SUVs in 50 mM KH_2_PO_4_/K_2_HPO_4_, 75 mM KCl, pH 7.4. Scale bars, 50 μm. (B) Following droplet production by a bulk shaking method (i), the oil–surfactant mixture was exchanged by the acidic oil–surfactant mix supplemented with 36 mM acetic acid, thus provoking the rapid assembly of a supported lipid bilayer at the droplet periphery (ii). Scale bar, 50 μm. (C) Sequential assembly of dsGUVs via a microfluidic mechanical splitting module by entrapping SUVs in a droplet with an oil–surfactant mix without supplementing acetic acid (i) and following the substitution of the oil phase by an acidic oil containing 36 mM acetic acid (ii). Alternatively, assembly of dsGUVs produced by microfluidic splitting can be achieved through the direct usage of an acidic oil containing 36 mM acetic acid as the continuous phase (iii). Scale bars, 25 μm. To minimize droplet coalescence under acidic oil conditions, the oil phase contained 3 wt % PEG-based fluorosurfactant and 10 mM Krytox and 35 mM acetic acid in HFE-7500. (D) Assembly of multicompartment dsGUVs by microfluidic mechanical splitting through post-production acidification. Droplets encapsulated two SUV populations: 1.5 mM SUVs composed of DOBAQ/DOPG/DOPC/Liss Rhod B labeled-DOPE (60/20/19.5/0.5 mol %) and 1 mM of Q_pa_DOPE/ATTO 488-labeled DOPE (99.5/0.5 mol %), both in 50 mM KH_2_PO_4_/K_2_HPO_4_, 75 mM KCl, pH 7.4. The oil–surfactant mixture was composed of 3 wt % PEG-based fluorosurfactant and 10 mM Krytox in HFE-7500 (Top). Following the production and collection of the dsGUVs, the oil phase was substituted by an acidic oil, initiating the rapid and selective fusion of DOBAQ SUVs to the droplet periphery (bottom). Scale bars, 25 μm.

Because the generation of dsGUVs relies on the generation of an emulsion, we exploited the capacity of the continuous oil phase to externally manipulate the pH of W/O droplets.^42,43^ Consequently, the acidification of the W/O droplets could be externally controlled after the co-encapsulation of the two SUV population and splitting of the droplets to prevent potential clogging of the small channels. To first evaluate this hypothesis, we produced dsGUVs encapsulating pH-sensitive SUVs in a well-buffered aqueous solution composed of 50 mM KH_2_PO_4_/K_2_HPO_4_ pH 7.4 and generated W/O droplets by the shaking method. For these experiments, the oil–surfactant mixture was supplemented with various concentrations of acetic acid, a small organic acid soluble in both the aqueous and the fluorinated oil phase. The shaking method allowed for a rapid prototyping of various lipid compositions, buffers, and surfactant–oil mixtures with minimal volume of constituents rather than directly applying microfluidics. In all cases, we kept a water to oil ratio of 1:2, with typical volumes of 10:20 μL, respectively (Table S1, Supporting Information note 1). Upon increasing the concentration of acetic acid up to 36 mM, we observed a significant recruitment and fusion of the pH-sensitive SUVs to the droplet periphery, while none or negligible recruitment was observed at lower concentration of acetic acid with these lipids and buffer composition (Figure 2A). Interestingly, by increasing the acid concentration, we observed a reduction of the droplet size. This was rationalized by a reduction of the interfacial tension, which favors the breaking up of large droplets due to the increase in ionic strength within the W/O droplets by the acid. This increase in ionic strength also promotes the concomitant adsorption of further ionic surfactant (i.e., Krytox) at the interface, which may further reduce the interfacial tension.^44^ Alternatively, we evaluated the possibility to initiate the fusion of pH-sensitive SUVs to the droplet periphery in a sequential manner, following the production of SUV-containing droplets. Toward this end, the preformed SUV-containing droplets were exposed to a surfactant–oil mixture supplemented with 36 mM acetic acid (Figure 2B). A rapid assembly of dsGUVs was observed upon the exposure to the acidic oil conditions. Again, we observed a reduction in size following the introduction of acid, where the reduction in surface tension promoted the breakup of large preformed droplets with the aid of mechanical stress upon oil substitution.

Following the successful assembly of dsGUVs under bulk conditions by applying a pH trigger from the oil phase, we evaluated if the use of an acidic oil could empower and facilitate the direct translation of this approach toward small channel geometry microfluidics. We observed that both approaches, either post-production acidification or the direct use of an acidic oil, enable the reliable production of dsGUVs by mechanical splitting (Figure 2C; video S2). Interestingly, due to their inherent and homogeneous small size, no significant difference in droplet size was observed before and after oil substitution, thus reinforcing the idea that larger droplets break up upon the reduction of surface tension and mechanical stresses. In addition, the use of an acidic oil still enables the selective recruitment of pH-sensitive SUVs to the droplet periphery to allow for the assembly of compartmentalized dsGUVs (Figure 2D). By using mechanical splitters, two population of SUVs were co-encapsulated within W/O droplets possessing an average diameter of 7.7 ± 0.9 μm (*n* = 212; Figure 2D top; Figure S9) and 8.5 ± 1.4 μm (*n* = 181; Figure 2D bottom; Figure S9) before and after the introduction of the acidic oil phase, respectively. Thus, the use of acetic acid—which is soluble in both the water phase and the fluorinated oil phase—can modulate the pH of the W/O droplets to mediate the assembly of dsGUVs encapsulating pH-sensitive SUVs.

**Figure 3:**
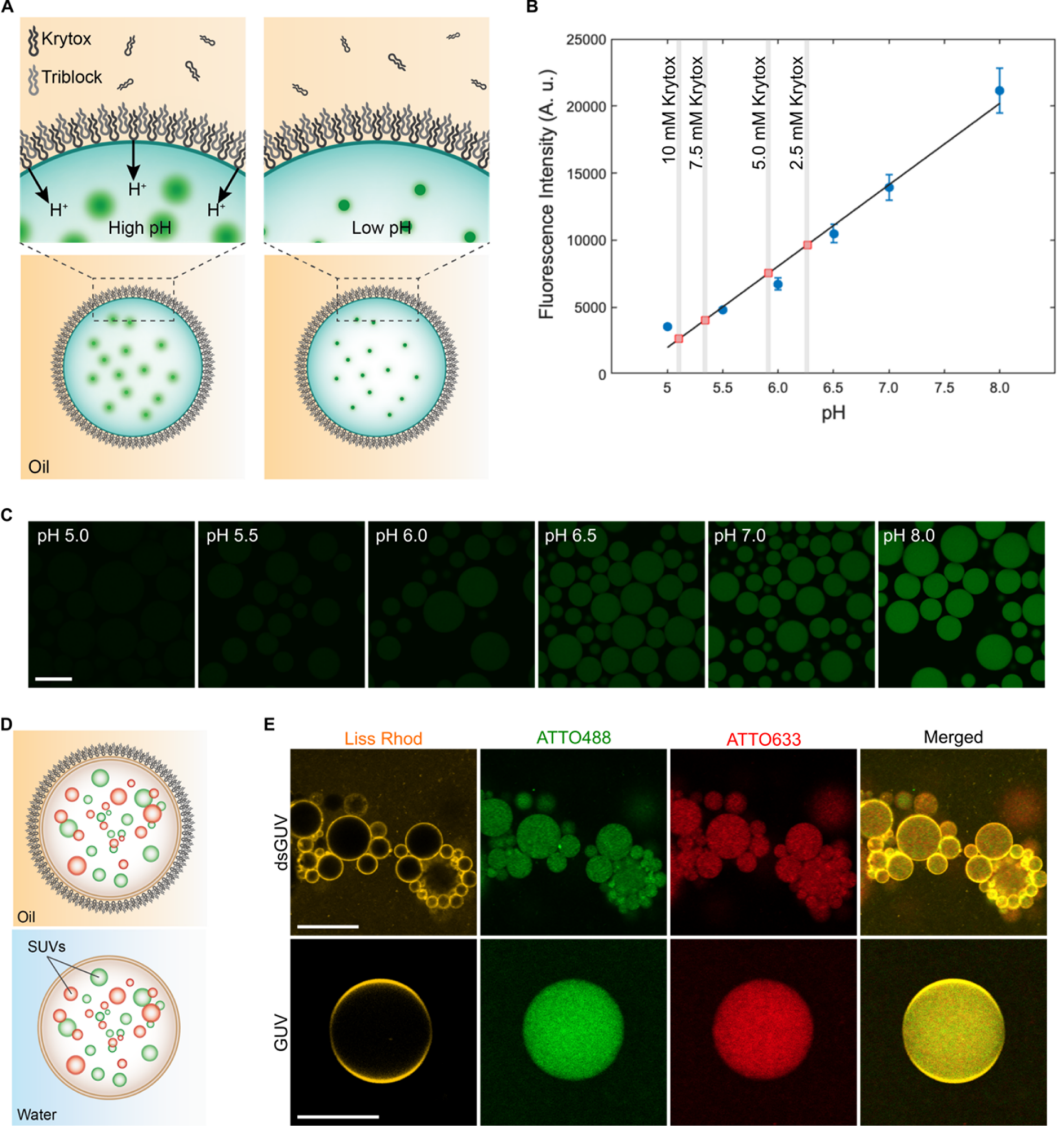
(A) Schematic representation of the fluorescein-based detection of concomitant acidification of the W/O aqueous core by the addition of Krytox in the oil–surfactant mixture. At physiological pH (left image), the fluorescence of fluorescein is maximal, while upon acidification, the fluorescence is diminished (right image low pH). (B) Calibration curve of the mean fluorescence intensity excited at 488 nm of W/O droplets encapsulating 1 μM fluorescein in 10 mM KH_2_PO_4_/K_2_HPO_4_ and 140 mM KCl at various pH values. W/O droplets were generated by manual shaking to produce the emulsion by employing an oil–surfactant mixture composed of 2.5 mM PEG-based florosurfactant and various Krytox concentrations (2.5; 5.0; 7.5; and 10 mM, represented by a vertical lines) in HFE-7500. A partitioning assay (see Supporting Information) detected a Krytox contamination of 46 μM into the PEG-based fluorosurfactant, which was considered negligible (Figure S8). The calculated pH of droplet population at each Krytox concentration is depicted by the red squares. Mean ± S.D are presented (*n* ≥ 50 droplets). (C) Representative CLSM images of the fluorescein-containing W/O droplets produced at various pH values. Scale bar: 100 μm. (D) Schematic representation of the generation of multicompartment dsGUVs through the co-encapsulation of different SUV populations and release under physiological conditions. I CLSM images presenting the self-assembly of compartmentalized droplet-stabilized GUVs achieved via shaking. (Top) dsGUVs generated after the encapsulation of three SUV populations: (1) 1.5 mM pH-sensitive SUVs composed of DODMA/DOPG/DOPC/DMG-PEG/Liss Rhod B PE (30/15/50.5/4/0.5 mol %), (2) 1 mM of redox-sensitive SUVs composed of Q_pa_DOPE/ATTO 488-labeled DOPE (99.5/0.5 mol %), and (3) 1 mM of negatively charged SUVs composed of DOPG/DOPC/ATTO633-labeled DOPE (30/69.5/0.5 mol %). All SUVs were prepared in 10 mM KH_2_PO_4_/K_2_HPO_4_, 140 mM KCl, pH 7.4. Acidification of the droplet lumen was achieved through the direct presence of Krytox in the oil–surfactant mixture composed of 2.5 mM PEG-based fluorosurfactant, 7.5 mM Krytox in HFE-7500. Scale bar, 25 μm. (Bottom) Released GUV under physiological conditions presenting the homogeneous distribution of the inner compartments. Scale bar, 10 μm.

The pH-mediated assembly of compartmentalized dsGUVs depends on two interconnected parameters: (1) the buffering capacity of the aqueous phase and (2) the p*K*_a_ of the pH-sensitive lipid. Because the use of Krytox, a fluorinated carboxylic acid,^45^ will lead to a concomitant acidification of the droplet lumen, we investigated if the use of either low pH citrate buffer or acetic acid in the oil phase could be omitted. Toward this end, we reduced the buffering capacity of the aqueous phase from 50 to 10 mM phosphate buffer and supplemented 140 mM KCl in order to match the osmolarity and buffering capacity of a standard phosphate buffer saline (e.g., 1× PBS). Note, in order to minimize potential interactions in between population of SUVs and the droplet periphery, KCl was favored over NaCl due to the reduced interaction of K^+^ ions with phospholipids.^46^ To probe the effect of Krytox on the pH of the droplets, fluorescein was encapsulated within W/O droplets stabilized by 2.5 mM PEG-based fluorosurfactant in HFE-7500 in the absence of Krytox. A partitioning assay of the PEG-based fluorosurfactant showed a minimal amount (∼46 μM) of Krytox impurity (Figure S10), which was considered negligible. Fluorescein has an intricate sensitivity to pH, and its fluorescence intensity decreases in acidic environment (Figure 3A).^47^ The pH of the water phase in the presence of fluorescein was varied by adjusting the KH_2_PO_4_/K_2_HPO_4_/K_3_PO_4_ ratio within the range of pH 5 to 8. Droplets were then imaged by CLSM, where the fluorescence intensity showed a linear correlation with the droplet’s inner pH (Figure 3B,C). Then, droplets containing 10 mM KH_2_PO_4_/K_2_HPO_4_ and 140 mM KCl at pH 7.4 were generated by supplementing various concentrations of Krytox (2.5; 5.0; 7.5, and 10 mM) to an oil–surfactant mixture containing 2.5 mM PEG-based fluorosurfactant in HFE-7500. Under these conditions, we observed a drastic reduction of the droplet pH, reaching 5.1 at 10 mM Krytox (Figure 3B). Importantly, under such acidic conditions, the DOBAQ-containing SUVs typically assemble to generate dsGUVs as previously observed herein when citrate buffer or acetic acid was employed. We confirmed the successful assembly of dsGUVs by encapsulating pH-sensitive SUVs in 10 mM KH_2_PO_4_/K_2_HPO_4_ and 140 mM KCl at pH 7.4 in W/O droplets with solely implementing 10 mM Krytox as the acid source (Figure S11; Table S1).

Along with the reduction of the buffering capacity, we produced SUVs incorporating other pH-sensitive lipids exhibiting a greater p*K*_a_ than DOBAQ, which would thus modulate their charge at higher pH. We incorporated DODMA, a synthetic pH-sensitive lipid possessing a p*K*_a_ of 7.6 once incorporated in lipid nanoparticles.^33^ Interestingly, we measured a p*K*_a_ of 8.2 for DODMA by a TNS assay when incorporated into SUVs, highlighting the impact of the lipid environment on the p*K*_a_ of the lipid (Figure S12).^48^ By substituting DOBAQ for DODMA, we showed the selective self-assembly of an endomembrane system incorporating different types of compartments with a reduced concentration of Krytox of 7.5 mM when 10 mM KH_2_PO_4_/K_2_HPO_4_ and 140 mM KCl at pH 7.4 were used as the aqueous phase (Figure 3D,E). Herein, functional SUVs, incorporating the redox-sensitive lipid Q_pa_-DOPE labeled with ATTO488, and a passive SUVs labeled with ATTO633 were all encapsulated within dsGUVs by a pH trigger. Moreover, zwitterionic SUVs (i.e., solely DOPC-containing SUVs) were also successfully encapsulated with our pH-mediated approach, thus expanding its potential for the generation of functional multicompartment synthetic cells (Figure S13). These results further highlight the potential of the pH-triggered assembly of dsGUVs to build multifunctional synthetic eukaryotes possessing stimuli-responsive compartments. To summarize, the use of an external source of acid (i.e., from the oil phase) can be employed to either trigger or directly assemble compartmentalized dsGUVs through simple emulsification (i.e., shaking) or microfluidic platforms when the aqueous phase possesses an important buffering capacity. External use of acid (i.e., acetic acid) can be omitted, when low buffering capacity of the aqueous phase is used. Herein, we showed that the intricate acidification by Krytox is sufficient to trigger the assembly of dsGUVs in buffer similar to 1× PBS. Moreover, the pH of assembly may be tuned based on the p*K*_a_ of the pH-sensitive lipid, hence offering additional control and flexibility to assemble compartmentalized dsGUVs.

### Usage of pH Improves the SUV to GUV Conversion

As opposed to conventional lipids, which are mostly zwitterionic in nature, pH-sensitive lipids grant the capacity to modulate the surface charge of SUVs in a relevant physiological pH window. This charge modulation was used to initiate the assembly of dsGUVs through the reduction of the droplet pH. One of the drawbacks of the charge-mediated assembly of GUVs, which is also related to its advantage, is the use of W/O droplets stabilized by fluorosurfactants. These droplets act as the molecular template, where their size dictates the final size of the GUVs, while providing important mechanical stability. To release the assembled GUVs into physiological conditions, the droplets must be destabilized, that is, the stabilizing surfactant must be removed. This destabilization process is achieved through the addition of an excess of small and poorly stabilizing surfactant such as 1*H*,1*H*,2*H*,2*H*-perfluoro-1-octanol (PFO), promoting droplet coalescence and hence the release of the GUVs into the water phase. For successful and effective release from the droplet, molecular interactions between the droplet interface and the lipids must be the lowest in order to minimize mechanical stresses. The typical use of Krytox and Mg^2+^ ions to recruit and fuse SUVs to the droplet periphery corresponds to a strong ionic interaction, which cannot be easily altered or dynamically modulated to maximize GUV production. Herein, we envision that the external modulation of the surface charge may facilitate and improve the release efficiency of dsGUVs to isolate free-standing GUVs.

**Figure 4:**
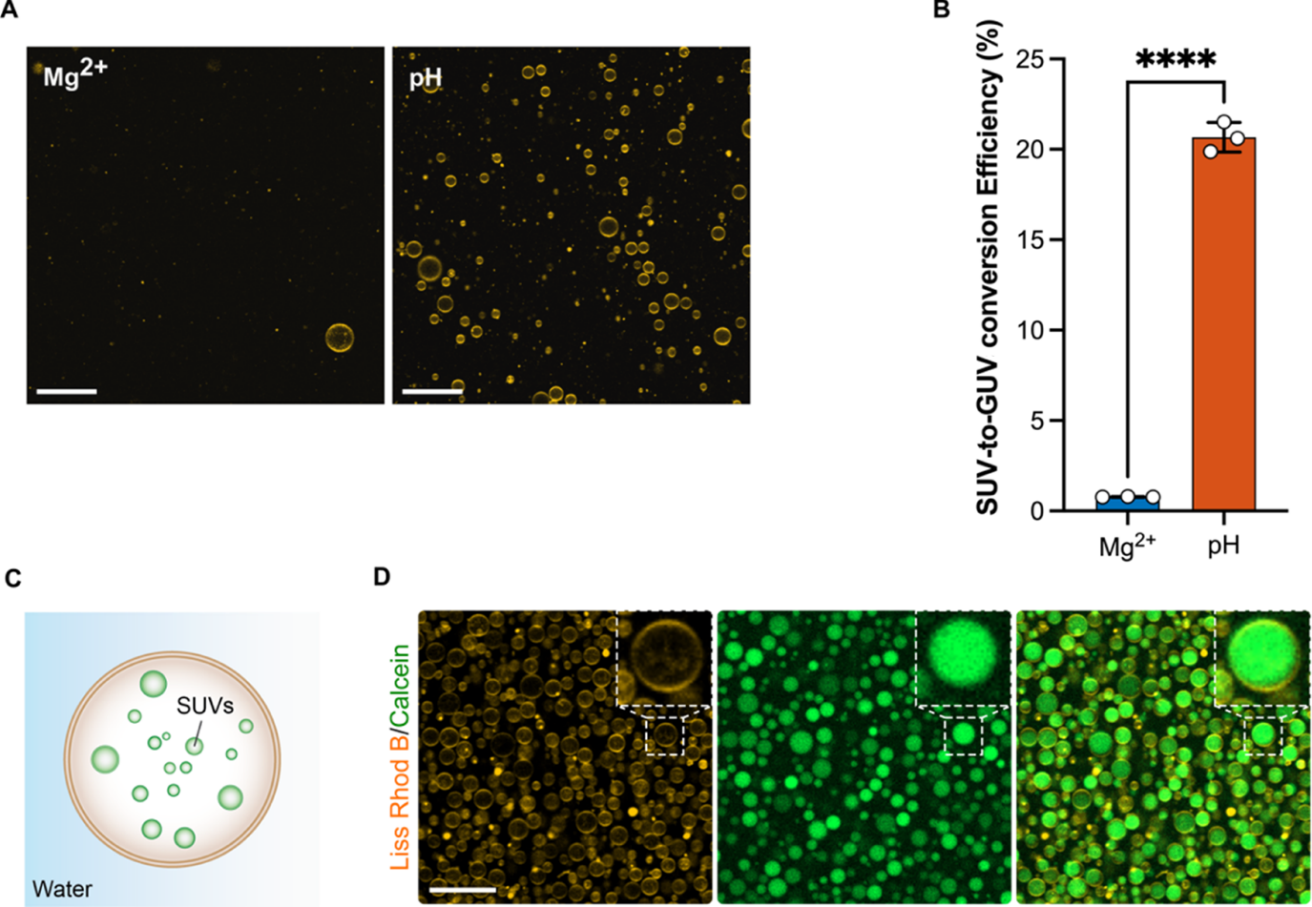
(A) Representative CLSM images of randomly selected locations within the observation chamber of released GUVs produced from precursor SUVs containing DOBAQ/DOPG/DOPC/DOPE/Liss Rhod B-labeled DOPE (30/20/39.5/10/0.5 mol %). GUVs were assembled with either the presence of 10 mM MgCl_2_ (referred as Mg^2+^) or at pH 5 (referred as pH) through the acidification of the W/O droplets from the oil phase by Krytox. In the case of Mg^2+^-mediated assembly, the aqueous phase was supplemented with 10 mM MgCl_2_ and 30 mM Tris buffer pH 7.4. In the case of the pH-mediated assembly, the aqueous phase was 10 mM KH_2_PO_4_/K_2_HPO_4_, 140 mM KCl, pH 7.4. W/O droplets were produced by manual shaking to achieve an emulsion. Droplets were stabilized by 2.5 mM of PEG-based fluorosurfactant and 10 mM Krytox in HFE-7500, with a water to oil ratio of 1:2. Scale bars: 50 μm. (B) SUV-to-GUV conversion efficiency of GUVs prepared by Mg^2+^- and pH-mediated assemblies as evaluated by fluorescence spectroscopy. Mean ± S.D. are presented (*n* = 3). Data were analyzed using an unpaired *t*-test. ****P < 0.0001. The SUV to GUV conversion efficiency corresponds to *N*_GUVs_/*N*_GUVs theoretical_, where *N*_GUVs_ is the total number of GUVs released from the emulsion and *N*_GUVs theoretical_ is the theoretical total number of GUVs possible to produce based on the number of SUVs within the W/O droplets. Lipid concentration of samples was measured by fluorescence according to the signal of Liss Rhod B labeled-DOPE contained in GUVs in a mixture of 1:1 isopropanol/buffer, with the aid of a calibration curve of the precursor SUVs in isopropanol/buffer presented in Figure S16. (C) Scheme presenting the formation of free-standing multicompartment GUVs under physiological conditions. (D) Multicompartment GUVs encapsulating 1 mM calcein-loaded Q_pa_DOPE SUVs (100 mol %). The GUVs were assembled by the pH-mediated approach from precursor SUVs composed of DOBAQ/DOPG/DOPC/DOPE/Liss Rhod B-labeled DOPE (30/20/39.5/10/0.5 mol %), employing mechanical splitter microfluidics for W/O droplet production to achieve higher release efficiency and improve polydispersity. Scale bar: 25 μm.

Toward this aim, we compared the release efficiency of dsGUVs produced by either the standard procedure using 10 mM Mg^2+^ ions^3,26,27^ or assembled by a pH trigger. For direct comparison, a SUV formulation, composed of DOBAQ/DOPG/DOPC/DOPE/Liss Rhod B-labeled DOPE (30/20/39.5/10/0.5 mol %) was produced. Importantly, this formulation possessed an optimized DOBAQ/DOPG ratio, which enable both the use of pH or Mg^2+^ ions to mediate the assembly. SUVs were encapsulated into the W/O droplet stabilized by 2.5 mM PEG-based fluorosurfactant and 10 mM Krytox in HFE-7500. For the Mg^2+^ ion-mediated assembly of dsGUVs, the aqueous phase was supplemented with 10 mM MgCl_2_ and 30 mM Tris buffer pH 7.4. We observed that without Mg^2+^ ions, SUVs did not fuse to the periphery, thus confirming no pH-mediated assembly in 30 mM Tris buffer when 10 mM Krytox was used (Figure S14). In the case of pH-mediated assembly, the aqueous phase was supplemented with 10 mM KH_2_PO_4_/K_2_HPO_4_, 140 mM KCl, pH 7.4 and employed the same oil–surfactant mixture. Following their production and incubation, dsGUVs were released with an osmotically matching buffer at pH 7.4. CLSM imaging revealed the striking increase in absolute number of released GUVs when pH was employed compared to Mg^2+^ (Figures 4A, S15). In the case of pH-mediated assembly, we postulated that the interactions between the Krytox and the lipids at the droplet interface were minimized due to the release at pH 7.4. At physiological pH, the surface charge of the dsGUVs becomes more negative, as the DOBAQ lipid became a zwitterion at pH > 4.35. This increase in negative charge leads to a reduction of the electrostatic interaction between lipids and Krytox at the droplet periphery and may even promote electrostatic repulsion. When dsGUVs were released at pH 5, a reduced number of GUVs was observed, which corroborate the presence of strong remaining interactions between Krytox and DOBAQ lipids at pH 5 compared to pH 7.4.

To further quantify the efficiency of GUV production between pH and Mg^2+^ ion-mediated assembly, we measured what we referred to as the SUV-to-GUV conversion efficiency (Figure 4B). The SUV to GUV conversion efficiency corresponds to the ratio *N*_GUVs_/*N*_GUVs,theoretical_, where *N*_GUVs_ is the measured number of GUVs released from the emulsion and *N*_GUVs,theoretical_ is the theoretical total number of GUVs possible to be produced using the provided lipids during the assembly. The number of produced and released GUVs was assessed by fluorescence spectroscopy in a 1:1 isopropanol/water mixture and compared to a calibration curve of the precursor SUVs in 1:1 isopropanol/water (Figure 4C), where the increase in fluorescence was associated with the presence of GUVs rather than lipid aggregates, as shown in CLSM images of Figure 4A. Here, the use of an organic solvent to solubilize the lipids into the water phase was essential in order to compare the calibration curve generated from SUVs and GUVs. Results revealed that the SUV-to-GUV production efficiency is 20-fold greater when a pH trigger was used over Mg^2+^ ions with an average SUV-to-GUV conversion efficiency of 20% and also achieved high conversion efficiency with commercially available PEG-based fluorosurfactant (Supporting Information note 2; Figures S15B and S16). When translated to a microfluidic platform to achieve a lower polydispersity, we observed a tremendous improvement in production efficiency, thus achieving a high yield generation of compartmentalized GUVs (Figure 4D,E). In summary, the pH-mediated assembly showed improved production efficiency compared to the standard Mg^2+^ ion-mediated assembly, while also empowering the generation of multicompartment GUVs.

### pH-Mediated Assembly of Multicompartment GUVs with an Actin-Cytoskeleton

Up to now, protein-encapsulated dsGUVs in the presence of Mg^2+^ ions showed poor production efficiency and depended on the isoelectric point P_i_ of the protein. Negatively charged cytosolic proteins, such as actin,^49^ may hinder the charge-mediated assembly of the lipid bilayer in the presence of high Mg^2+^ ion concentration (i.e., 10 mM). To palliate this issue, microfluidic platforms incorporating pico-injectors^50^ can be used to sequentially reconstruct an actin cytoskeleton,^3^ but release of actin-containing dsGUVs remained challenging.

**Figure 5:**
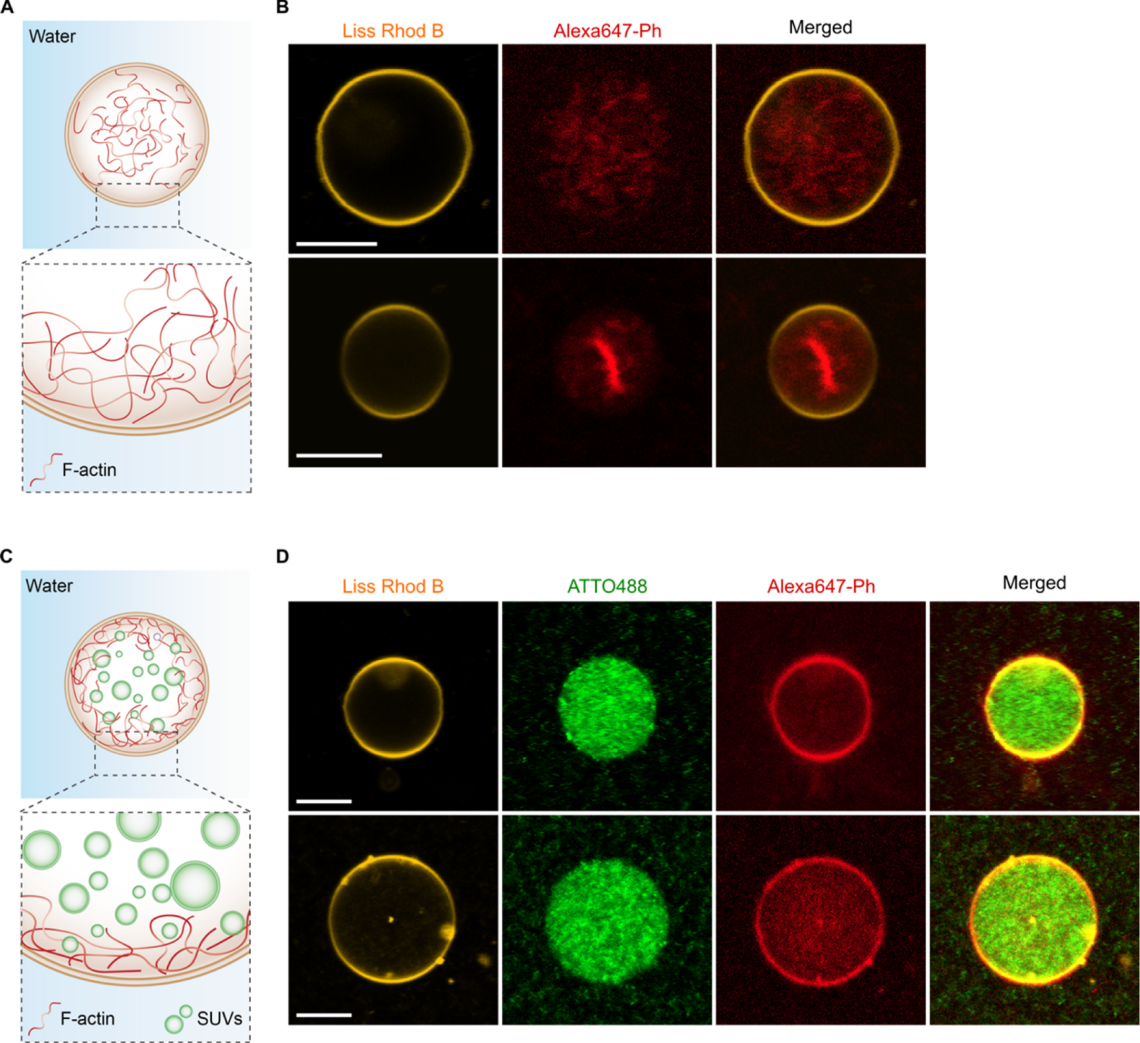
Reconstruction of an F-actin cytoskeleton in the absence and presence of an additional endomembrane system inside a synthetic eukaryote assembly through a pH trigger within a W/O droplet and release under physiological conditions. (A) Scheme presenting the encapsulation of F-actin within a free-standing GUV assembled by a pH trigger presenting the homogeneous distribution of F-actin within the lipid vesicle. (B) CLSM images of two representative cases of free-standing GUVs depicting either the relatively homogeneous distribution of the F-actin filaments or the confinement of the actin bundles at the center of the vesicles. Prior to their release, the GUVs were assembled by co-encapsulating pH-sensitive SUVs containing DOBAQ/DODMA/DOPG/DOPE/DOPC/DMG-PEG/Liss Rhod B-labeled DOPE (15/15/15/10/40.5/4/0.5 mol %), and 5 μM of F-actin labeled Alexa647-phalloidin was encapsulated in a surfactant-stabilized W/O droplet produced by vortexing. Scale bars: 10 μm. (C) Scheme presenting the encapsulation of both F-actin and SUVs imitating an endomembrane system within a free-standing GUV assembled by a pH trigger. (D) Representative CLSM images of two free-standing GUVs depicting a homogeneous distribution of the negatively charged inner compartment within the vesicle, whereas F-actin preferentially accumulates at the periphery of the lipid vesicle. The GUVs were assembled by supplementing 1 mM of negatively charged SUVs composed of DOPG/DOPC/ATTO488-labeled DOPE (30/69.5/0.5 mol %) to the mixture presented in (B) and encapsulating within surfactant-stabilized W/O droplets produced by vortexing. Scale bars: 10 μm.

Toward this goal, we applied the pH-triggered assembly of GUVs to palliate the needs of high Mg^2+^ ions and poor production efficiency and also to evaluate the potential of our method to co-encapsulate proteins and compartment. In a first step, we co-encapsulated pH-sensitive SUVs with actin filaments (F-actin) in a W/O emulsion generated by shaking. Upon acidification of the droplets by Krytox, we observed the recruitment and fusion of SUVs at the periphery, while the F-actin remained within the droplet lumen (Figure S17). The F-actin network was homogeneously distributed within the droplet lumen due to reduced interactions in between the F-actin and Krytox at low concentration of Mg^2+^. Note that a leakage of the Liss Rhod B-labeled DOPE lipids into the fluorinated oil phase under these experimental conditions was observed. The leakage is attributed to the strong stochiometric association of rhodamine B to Krytox, which affects the retention of dyes within the W/O droplet as a function of salt concentration, buffer, and Krytox concentration.^27,51,52^ Following their production, the F-actin-containing dsGUVs were successfully released under physiological conditions (Figure 5A,B). We observed that the released GUVs were typically smaller in size compared to the corresponding dsGUVs, suggesting two possible case of figures: first, large vesicles containing F-actin may hardly tolerate the bulk release process, leading to release of intraluminal F-actin into the aqueous buffer which was visible by CLSM at the bottom of the observation chamber (Figure S18). Second, vesicles may shrink due to slight changes in osmotic pressure.

Our results show that the produced GUVs typically retain their homogeneous distribution of F-actin (Video S3), or in few cases, exhibited a further accumulation of F-actin toward the center of the vesicle, as previously reported by Weiss and co-workers.^3^ This interesting difference and improvement in the assembly can be first explained by the production method (pH-triggered, choice of PEG-based fluorosurfactant, and reduced Mg^2+^), but also by the introduction of additional electrostatic interactions resulting from changes in pH. Actin monomers possess an isoelectric point at pH ≈ 5.4,^53^ meaning that upon acidification by Krytox, actin will possess a slight excess of positive charges. In this case, both the SUVs and the F-actin would possess an excess of positive charges, hence minimizing their interaction with each other. Moreover, the pH-sensitive SUVs are expected to diffuse more rapidly to the negatively charged droplet interface owing to their smaller size. By assuming a 3D Brownian motion of 100 nm SUVs encapsulated within a 15 μm diameter W/O droplet and by applying the Stoke–Einstein law of diffusion for a spherical particle, an SUV located at the center of the droplet would reach the droplet interface in ≈400 ms, corresponding to a diffusion coefficient *D*_SUV_ of 24 μm2/s (Supporting Information note 3). F-actin, on the other hand, possesses a translational diffusion coefficient *D*_actin_ typically inferior to 1 μm^2^/s when located within two thin walls.^54^ Even though *D*_actin_ is expected to be higher in unbound fluids (i.e., within the W/O droplet), the diffusion of F-actin is greatly affected by its length, which in our case, will significantly be reduced by the presence of long micrometer-scaled filaments. Consequently, SUVs will diffuse faster than the F-actin at pH ≈ 5 toward the droplet interface to promote the assembly of dsGUVs.

Interestingly, when compartments were co-encapsulated with F-actin and pH-sensitive SUVs in W/O droplets, we observed accumulation of F-actin at the vesicle’s periphery, even in the absence of any (bio)chemical linkers.^55^ In addition, the compartments showed a homogeneous distribution within the droplet-stabilized vesicle (Figure S19) and after their release under physiological conditions (Figure 5C,D). This behavior is associated with the depletion effect, where F-actin organized itself at the vesicle periphery in the presence of compartments. In this spatial configuration, the entropy of the SUVs is maximized, thus corresponding to the most thermodynamically favored structure of the system. Similar observations were previously reported for large particles, which spontaneously adsorbed at the vesicle periphery when co-encapsulated with smaller negatively charged particles.^56,57^

## Summary and Outlook

In summary, the usage of pH as an internal or external trigger to activate the charge-mediated assembly of dsGUVs allows for the reconstruction of an endomembrane system in either a bulk assembly or by microfluidics. To achieve a successful formation of GUVs, the method relies on two important criteria: (1) slightly negatively charged SUVs can be efficiently entrapped and (2) the use of a pH-sensitive lipid. Moreover, the assembly is dictated by the apparent p*K*_a_ of the pH-sensitive SUVs, which could then be readily optimized through chemical synthesis of desired lipids, where p*K*_a_ of synthetic lipids was extensively investigated and tuned in the past decade.^30,58^ By introducing a pH-sensitive motif, the surface charge of the GUVs can be modulated, which is the origin of a fundamental improvement in a total number of GUVs produced from the droplet-stabilized approach. Besides improving the production efficiency, the use of pH has empowered the reconstruction of an F-actin cytoskeleton with or without an endomembrane system. This corresponds to a very basic advancement in bottom-up synthetic biology employing solely lipid-based vesicles. Interestingly, we observed a drastic change in the behavior of F-actin in the presence of compartments due to the depletion effect, highlighting the possibility to observe and investigate emergent properties resulting from the combination of different cellular modules. Still, further experiments and optimization would be required to investigate the behavior of F-actin in the presence of molecular crowder, such as SUVs, inside GUVs in greater detail. Additionally, moving from F-actin to actin monomers would be more relevant. In that sense, compartments could be engineered to regulate actin polymerization and ultimately recreate cellular motility inside synthetic eukaryotes. The method presented herein could thus catalyze the assembly of more specialized synthetic eukaryotes for biotechnological and medical applications. By entrapping various stimuli-responsive compartments, minimal cross-reactivity and control over the release sequence could be embedded within a single synthetic eukaryote. The assembly of such an endomembrane system within a micron-scale carrier could be engineered to release therapeutics through the vascular walls,^59^ while enabling improved functionality, reduced passive leakage, cross-reactivity, and flexibility to name a few.

## Materials and Methods

ATTO488-labeled 1,2-dioleoyl-*sn*-glycero-3-phosphoethanolamine (ATTO488-labeled DOPE) was purchased from ATTO TEC (Germany). 1,2-Dieoleyloxy-3-dimethylaminopropane (DODMA) was synthetized as described elsewhere.^33^ Quinone proprionic acid-linked-1,2-dioleoyl-*sn*-glycero-3-phosphoethanolamine (Q_pa_DOPE) was prepared as reported elsewhere.^60^ All other lipids were bought from Avanti Polar Lipids as solution in chloroform and used without further purification. All lipids were stored at −20 °C until needed. The 008-PEG-based fluorosurfactant, also referred to as commercially available PEG-based fluorosurfactant (CFS), was bought from Ran Biotechnology, Inc. (MA, USA). All chemicals were bought from Sigma-Aldrich (Germany) and were used without further purification. Bovine serum albumin (BSA)-coated glass slides (25 × 60 mm^2^ and 18 × 18 mm^2^) were prepared by drop-coating a solution of 1 mg/mL BSA in 1× Dulbecco’s PBS (DPBS) for 15 min, dried, and rinsed with Milli-Q water. The BSA-coated glass slides were directly used to assemble an observation chamber through the usage of double-sided sticky tape.

### General Procedure for Preparation of SUVs

SUVs were prepared by lipid film hydration, followed by extrusion. Lipids from stock solutions in chloroform were mixed to the desired molar ratio in a glass vial. Chloroform was evaporated by blowing with a gentle stream of nitrogen to obtain a thin lipid film. To remove residual traces of organic solvent, the vial was desiccated under vacuum for a period of 2 h. The dry lipid film was then rehydrated through the addition of a solution of 10 mM KH_2_PO_4_/K_2_HPO_4_, 140 mM KCl, pH 7.4 (if not mentioned otherwise), at a final lipid concentration of 6 mM. The lipid film was swelled for 20 min and vortexed 30 s to trigger the rapid formation of multilamellar liposomes. The lipid suspension was extruded through a 100 nm polycarbonate track-etch membrane (Whatman), 21 times at a temperature at least 5 °C above the *T*_m_ of the lipids, 25 °C in most of our case, with a miniextruder (Avanti Polar Lipids, Inc.). The resulting SUVs were stored at 4 °C until needed for up to 3 days or used immediately for generating dsGUVs.

### Calcein-Loaded Q_pa_DOPE SUVs

5 mg of Q_pa_DOPE was dissolved in 5 mL of chloroform in a round bottom flask and evaporated under vacuum with a rotary evaporator for 1 h. The dried lipid film was re-hydrated with a solution of 50 mM calcein dissolved in 50 mM KH_2_PO_4_/K_2_HPO_4_, 75 mM KCl, pH 7.4 to a final lipid concentration of 1 mg/mL. The lipid film was aged for 1 h with occasional vortexing every 15 min, followed by five cycles of freeze–thawing in a dry ice/acetone bath. The lipid suspension was extruded through a 100 nm polycarbonate track-etch membrane (Whatman), 21 times at room temperature with a miniextruder (Avanti Polar Lipids, Inc.). Following extrusion, un-encapsulated calcein was removed by spin column filtration. Briefly, Sephadex-G50 resin fine (GE healthcare Bioscience) was swelled for at least 3 h in 50 mM KH_2_PO_4_/K_2_HPO_4_, 75 mM KCl, pH 7.4. The resin was added over a glass wool-plugged 2 mL syringe until the resin completely filled the syringe and then compacted by centrifugation (2 min, 1000*g*). 200 μL of liposomal solution was added to the column and centrifugated for 10 min at 50 g. Eluted calcein-loaded liposomes were then expelled and collected from the column by centrifuging 2 min at 1000*g*. The resulting unilamellar calcein-loaded SUVs were stored at 4 °C until needed for up to 7 days. Note that in the case of calcein-loaded SUVs, extrusion was performed through a 100 nm membrane to facilitate and improve the purification step achieved by size exclusion.

### Synthesis of Triblock-co-polymer Fluorosurfactant

The synthesis of a fluorosurfactant, composed of PFPE–PEG_1500 MW_–PFPE, was adapted from the work of Scanga and co-workers.^61^
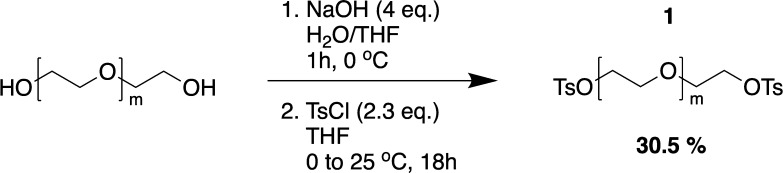


#### PEG_1500_ Ditosylate (**1**)

Sodium hydroxide (13.32 g, 333 mmol, 4.0 equiv) was dissolved in water (103 mL) under ice cooling. Tetrahydrofuran (200 mL) was added to the solution, and the mixture was allowed to cool down to 0 °C. PEG 1500 (125 g, 83.3 mmol, 1.0 equiv) was added in small portions, so that the temperature did not rise above 5 °C. Afterward, the mixture was allowed to warm up to room temperature and stirred for 1 h. After the reaction mixture was cooled to 0 °C again, *p*-toluenesulfonic acid (36.4 g, 191 mmol, 2.3 equiv) dissolved in tetrahydrofuran (220 mL) was added dropwise and care was taken that the temperature did not rise above 5 °C. The reaction mixture was stirred for 18 h, allowing the temperature to rise to room temperature. The organic layer was separated, and the solvent was removed under reduced pressure. After dissolving the crude product in ethyl acetate (750 mL), the solution was washed with 90 vol % brine (100 mL). The solution was dried over magnesium sulfate and filtered. Afterward, the solvent was removed under reduced pressure. The tosylated product **1** was received as a white solid (46.0 g, 25.4 mmol, 30.5%). ^1^H NMR (400 MHz, CDCl_3_): δ 2.385 (s, 6H, H6), 3.578 (m, H3, H2), 4.090 (t, *J* = 4.4 Hz, 4H, H1), 7.283 (d, *J* = 4.0 Hz, 4H, H5), 7.729 (d, *J* = 4.0 Hz, 4H, H4).
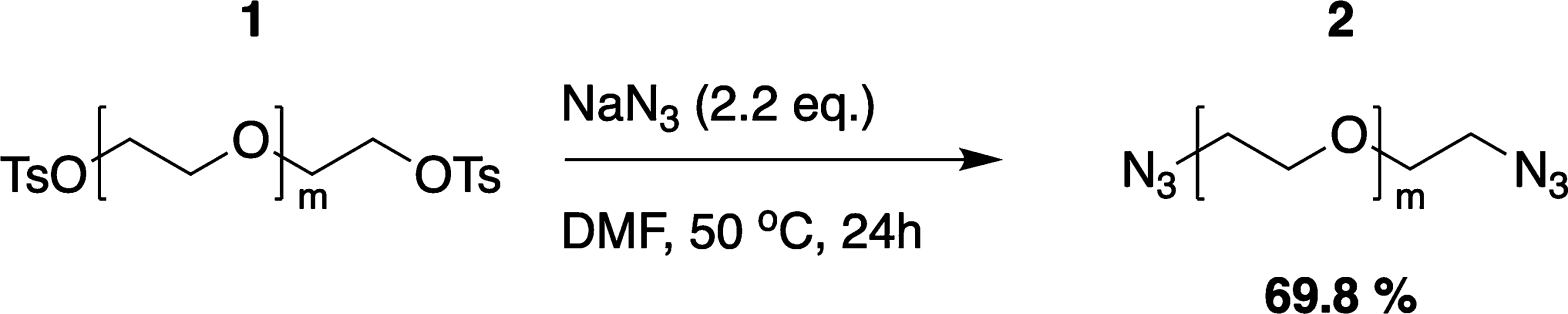


#### PEG_1500_ Diazide (**2**)

Compound **1** (20.0 g, 11.05 mmol, 1.0 equiv) was dissolved in dimethylformamide (30 mL), and sodium azide (1.58 g, 24.3 mmol, 2.2 equiv) was added. The mixture was stirred first for 90 min at room temperature, followed by 18 h at 50 °C. As the reaction progresses, the mixture becomes more and more turbid. The mixture was filtered, and the solvent was removed by co-evaporation with toluene under reduced pressure. Afterward, the crude product was resuspended in ethyl acetate (200 mL), filtered, and washed with 90 vol % brine (50 mL). The obtained solution was dried over magnesium sulfate and then filtered, and the solvent was removed under reduced pressure. The diazide substituted product **2** was received as a white solid (11.9 g, 7.71 mmol, 69.8%). ^1^H NMR (400 MHz, CDCl_3_): δ 3.379 (t, *J* = 5.2 Hz, 4H, H1), 3.634 (m, H2, H3).
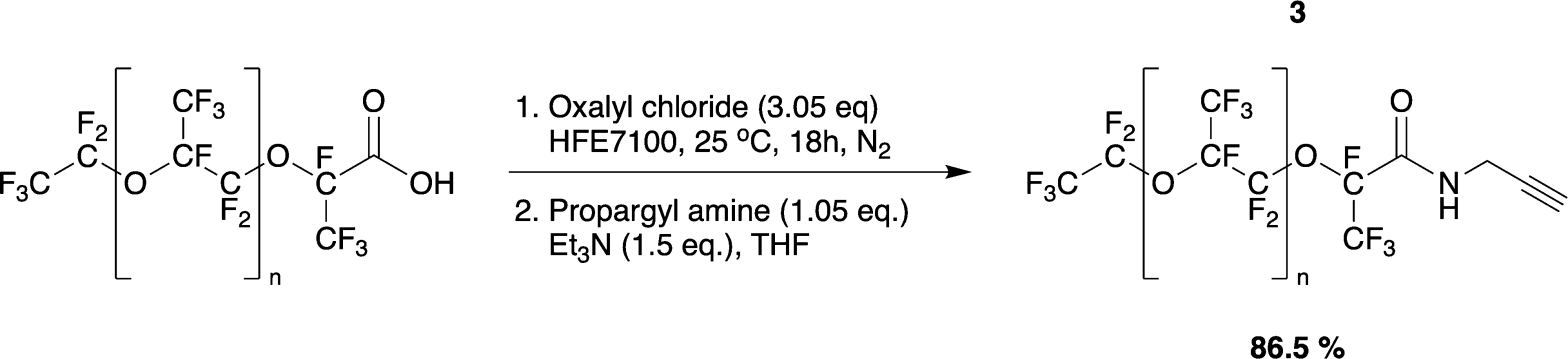


#### Propargyl PFPE_7000_ (**3**)

In the first step, PFPE acid (Krytox FSH, 90.23 g, 12.89 mmol, 1.0 equiv) was filled into a flame-dried flask, degassed under reduced pressure, and dissolved in HFE 7100 (150 mL). Oxalyl chloride (3.4 mL, 39.4 mmol, 3.05 equiv) was added, and the reaction mixture was refluxed for 18 h, during which the reaction mixture became cloudy. The solvent and excess of oxalyl chloride were removed under reduced pressure, collecting the removed material in a liquid nitrogen cooling trap. In a second step, the intermediate product was dissolved in HFE 7100 (90 mL). The reaction vessel was equipped with a dropping funnel under nitrogen counterflow. The funnel was loaded with propargyl amine (870 μL, 13.5 mmol, 1.05 equiv), trimethylamine (2.7 mL, 19.3 mmol, 1.5 equiv), and tetrahydrofuran (35 mL). The propargyl amine solution was added dropwise, and the reaction mixture was stirred for 18 h. The solvent and other volatile reagents were removed under reduced pressure. The obtained crude product was dissolved in HFE 7100 and filtered. After removing the solvent under reduced pressure, the propargyl derivative **3** was received as an orange oil (78.5 g, 11.15 mmol, 86.5%). As NMR solvent, a mixture of C_6_F_6_:C_6_D_6_ 88:12 was used. ^1^H NMR (400 MHz, C_6_D_6_): δ 2.186 (t, *J* = 2.8 Hz, 1H, H3), 4.135 (t, *J* = 2.8 Hz, 2H, H1), 6.602 (s, 1H, NH).
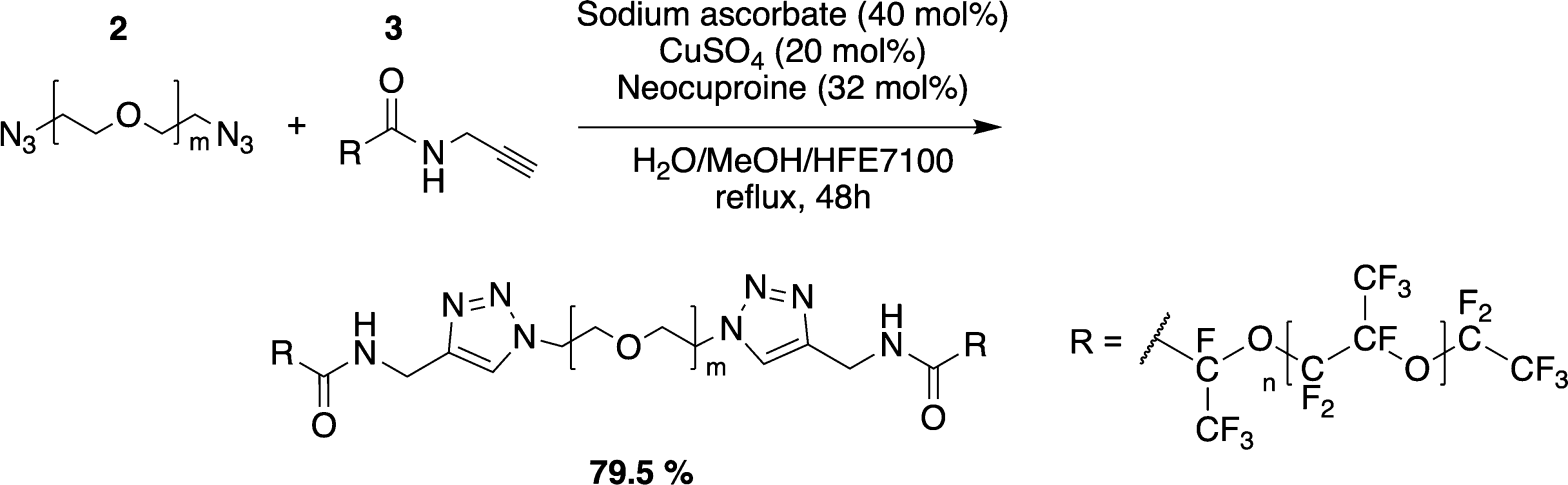


### PFPE_7000_–PEG_1500_–PFPE_7000_ Triazole-Linked Triblock Fluorosurfactant (SynFS)

Compound **3** (2.31 g, 1.49 mmol, 1.05 equiv), sodium ascorbate (112 mg, 568 μmol, 40 mol %), copper(II) sulfate pentahydrate (70.9 mg, 284 μmol, 20 mol %), and neocuproine (94.8 mg, 455 μmol, 32 mol %) were dissolved in water (15 mL) and methanol (15 mL). Propargyl PFPE 7000 (20.0 g, 2.84 mmol, 2.0 equiv) was dissolved in HFE 7100 (30 mL) and added to the aqueous solution. The reaction mixture was refluxed for 48 h. The reaction mixture was cooled to room temperature. Afterward, it was transferred in a separation funnel and carefully overlaid with methanol (15 mL). The two-phase system was swiveled without mixing. The methanol layer became orange and was removed after saturation. This procedure was repeated until the methanol phase did not become orange anymore. If the volume of fluorinated oil phase was decreased as much that the swiveling was not efficient anymore, HFE 7100 was added. Afterward, the fluorinated phase was dried over magnesium sulfate. The crude product was first filtered through Celite and then through a 0.45 μm PFTE syringe filter. The solvent was removed under reduced pressure. The triblock copolymer surfactant was received as a highly viscous substance (17.6 g, 1.13 mmol, 79.5%). ^1^H NMR (400 MHz, C_6_D_6_): δ 3.637 (m, H3), 3.970 (s, 4H, H2), 4.588 (s, 4H, H1), 4.678 (m, 4H, H6), 7.976 (s, 2H, H4), 8.433 (s, 2H, NH). The self-synthetized fluorosurfactant will be referred to as SynFS.

### Microfluidic Device Fabrication

Microfluidic devices were fabricated using polydimethylsiloxane (PDMS) and soft lithographic procedure. All microfluidic devices were designed using the computer-aided design (CAD) software QCAD-pro (RibbonSoft, Switzerland). Briefly, a thin layer of negative photoresist SU8-3005 (MicroChem, USA) was spin-coated (Laurell Technologies Corp., USA) at 1000 rpm on a 2 in. silicon wafer to produce a uniform 10 μm think layer. Then, wafers were soft-baked on a hot plate at 65 °C for 1 min and then ramped and held at 95 °C for 3 min. The CAD designs were directly exposed to the photoresist through a Tabletop Micro Pattern Generator μPG 101 (Heidelberg Instruments, Germany) employing the writing mode II. The exposure conditions were set to 70 mW for the output laser power and 25% for the pixel pulse duration. Following the exposure, the wafers were baked on a hot plate at 65 °C for 1 min and then ramped and held at 95 °C for 5 min. Next, mr-DEV 600 (MicroChemicals, Germany) was used to remove the unexposed SU8 resists from the wafer and hard baked in an oven at 150 °C for 15 min. After baking, the wafer was placed in a Petri dish and served as a mold for downstream PDMS fabrication. PDMS (Sylgard 184, Dow Corning, USA) was well mixed with the curing agent at a 10:1 ratio, degassed, poured onto the wafer, and cured for 2 h at 65 °C in an oven. The PDMS was then cut and carefully peeled off from the mold. Holes, serving as inlets and outlets, were generated using a 0.5 mm biopsy punch (Harris Uni-Core, Ted Pella, Inc.). Following punching, holes in the PDMS were cleaned with isopropanol and pressurized nitrogen gas to remove residual PDMS particles. Glass slides (24 × 60 mm^2^, Carl Roth, Germany) were sequentially cleaned using heptane and isopropanol and thoroughly dried with pressurized nitrogen gas. To bind the PDMS to a coverslip, the device and a clean glass slide were activated using an oxygen plasma (TePla, Germany; 0.4 mbar, 200 W, 35 s exposure) and brought in contact with gentle pressure. To strengthen the attachment between the PDMS and the glass slide, devices were heated 1 h at 65 °C in an oven. Sigmacote (Sigma-Aldrich, Germany) was passed through the channels to render their surface hydrophobic.

### General pH-Mediated Assembly of Multicompartment GUV in Bulk

Initially, we prepared an aqueous solution containing two populations of SUVs; 1.5 mM of pH-sensitive SUVs composed of DOBAQ/DOPG/DOPE/DOPC/DMG-PEG/Liss Rhod B DOPE (30/20/6/39.5/4/0.5 mol %) in 10 mM KH_2_PO_4_/K_2_HPO_4_, 140 mM KCl, pH 7.4, and 1 mM of the SUVs to be entrapped, typically composed of DOPG/DOPC/ATTO488 labeled-DOPE (30/69.5/0.5 mol %) in 10 mM KH_2_PO_4_/K_2_HPO_4_, 140 mM KCl, pH 7.4. Then, an oil–surfactant mixture composed of 2.5 mM of SynFS and 10 mM PFPE–carboxylic acid (Krytox-157FSH, MW 7000–7500 g/mol, DuPont, Germany) in HFE-7500 (3M, Germany) was prepared and filtered through a 0.22 μm polycarbonate filter. As an alternative, the SynFS may be substituted by commercially available PFPE–PEG fluorosurfactant (Ran Biotechnologies, Inc.) at a final concentration of 1.4 wt/wt %. However, we observed a substantial decrease in release efficiency when pH was employed as a strategy of self-assembly as opposed to Mg^2+^. Then, 50 μL of the SUVs containing aqueous solution was layered on top of 100 μL of the oil–surfactant mixture and vortexed vigorously for 30 s. The visible and persistent milky-like emulsion located above the excess of oil phase indicates the formation of stable W/O droplets. In these conditions, the pH-sensitive SUVs were preferentially recruited at the droplet periphery and fused to from a spherical supported lipid bilayer, resulting in the entrapment of the other SUVs population within the formed droplet-stabilized GUV. To release the GUVs from the oil–surfactant phase, 75 μL of 1× DPBS (2.7 mM KCl, 1.47 mM KH_2_PO_4_, 8.1 mM Na_2_HPO_4_, 138 mM NaCl, Gibco, ThermoFisher Scientific) or an osmolarity-matched buffer was slowly added on top of the droplet emulsion. In order to destabilize the droplets, 75 μL of PFO acting as a destabilizing agent was slowly added on top of the aqueous phase. The sample was gently rolled and left at 4 °C overnight. After incubation, the milky emulsion disappeared and led to a transparent aqueous layer on top of the oil–surfactant mixture. The top aqueous layer, containing the released GUVs, was carefully collected with a micropipette, while avoiding the collection of oil, and either directly transferred into a BSA-coated observation chamber for direct fluorescence imaging or stored in a new test tube at 4 °C until needed for up to 3 days. Although direct release is possible, we observed that the release efficiency (i.e., the absolute number of GUVs per mL) was significantly improved by storing the dsGUVs at 4 °C for a period of at least 2 h, and ideally overnight, prior to the addition of the release buffer and the destabilizing agent. As a general rule, the pH-mediated assembly was optimized to a water: oil ratio of 1:2, while we noted a maximal release efficiency when a water/release buffer (and PFO) of 1:1.5 ratio was used. The assembly can easily be scaled up and down, based on the experimental needs, without significant loss in release efficiency as long as these ratios are kept constant.

### pH-Mediated Assembly of Multicompartment GUV by Microfluidics

Stable W/O droplets were generated at the flow-focusing T-junction by employing the oil–surfactant mixture and the aqueous phase described above as continuous and disperse phase, respectively. Fluids were introduced within the microfluidics via PTFE tubing (0.3 mm I.D., 0.6 mm O.D., BOLA Tubing) with a pressure controller (OB1 Mk3; Elveflow). Pressure was adjusted with the ESI software (v3.01.13; Elveflow). Typical pressure for flow-focusing T-junction was between 500 and 750 mbar for both the disperse and continuous phase, while a pressure of 2 bar for both disperse and continuous phase was needed for the mechanical splitters. The droplet production was assessed through an axio table-top inverted microscope (Zeiss, Germany) via a 10× LD-A-Plan objective (NA = 0.25) equipped with a high-speed camera EoSens CL (Mikrotron GmbH). Following the production of the droplets, the resulting dsGUVs were incubated for a period of at least 2 h at 4 °C, or ideally overnight, prior to their release. The release procedure remains the same as for the bulk production described above. In general, the volume of the droplet produced was in the range of 100 μL.

### Actin Preparation

Actin was purified from acetone powder from New Zealand white rabbit skeletal muscle, based on the method of Pardee and Aspudich,^62^ and modified according to Kron and co-workers,^63^ and stored in 2 mM Tris–HCl, 0.2 mM CaCl_2_, 0.2 mM ATP, 0.005 % wt/v NaN_3_, and 0.2 mM DTT at pH 8, at −80 °C. Actin monomers were labeled with phalloidin-Alexa647 (Sigma-Aldrich) by mixing 72 μL actin monomers with 10 μL of 10× actin polymerization buffer (20 mM Tris-HCl, pH 8.0, 500 mM KCl, 20 mM MgCl_2_, 10 mM NaATP) and 18 μL of 2× actin buffer [AB; 50 mM imidazole, pH 7.4, 50 mM KCl, 2 mM ethylene glycol tetraacetic acid (EGTA), 8 mM MgCl_2_]. The actin monomers were left at room temperature to polymerize for 30 min. Subsequently, 10 μL (20 μL in MeOH, evaporated to ∼10 μL) of phalloidin-Alexa647 (10 units) was added to the solution. The resulting phalloidin-Alexa647-labeled F-actin was stored at −80 °C until needed.

### F-Actin Encapsulation with and without Inner Compartment into GUVs

The reconstruction of an F-actin cytoskeleton within GUVs in the presence or absence of an inner compartment was achieved by co-encapsulation of pH-sensitive SUVs and polymerized F-actin into W/O droplets stabilized by a PEG-based fluorosurfactant in the presence of a low amount of Mg^2+^ ions. First, pH-sensitive SUVs containing DOBAQ/DODMA/DOPC/DOPG/DOPE/DMG-PEG/Liss Rhod B-labeled DOPE (15/15/44.5/15/6/4/0.5 mol %) were produced by lipid film hydration with AB (25 mM imidazole, 25 KCl, 1 mM EGTA, 4 mM MgCl_2_, pH 7.4) at a concentration of 6 mM of lipid and extruded as described above in the general procedure. Inner compartments composed of DOPC/DOPG/ATTO488-labeled DOPE (79.5/20/0.5 mol %) were also prepared by lipid film hydration in AB, at a final concentration of 6 mM of lipid, and extruded. To assemble the synthetic eukaryote possessing an F-actin cytoskeleton, pH-sensitive SUVs were gently mixed with F-actin labeled with phalloidin-Alexa647 in AB at a final concentration of 1.5 mM and 5 μM in a 1.5 mL Eppendorf. Alternatively, 1 mM of inner compartments may be supplemented into the aqueous phase in order to recreate an endomembrane system. Then, 50 μL of the SUVs F-actin mixture was layered onto 100 μL of a fluorous phase composed of a 2.5 mM PEG-based fluorosurfactant and 2.5 mM Krytox in HFE-7500 and rapidly emulsified by vortexing to generate droplet-stabilized GUVs. The resulting dsGUVs were stored at 4 °C for 2 h prior to their release. To release the dsGUVs under physiological conditions, 75 μL of AB was added to the Eppendorf, followed by 75 μL of PFO, and left undisturbed at 4 °C until complete disappearance of the milky emulsion is achieved. Free-standing GUVs were then imaged by CLSM in a sealed BSA-coated observation chamber.

### Confocal Fluorescence Microscopy

Confocal fluorescence imaging was performed on a Zeiss LSM 800 confocal microscope (Carl Zeiss AG, Germany) with a 20× (NA = 0.8) Plan-Apochromat air objective (Carl Zeiss AG, Germany). The pinhole aperture was set to one Airy Unit, and experiments were performed at room temperature. A digital offset of 2500 (16 Bits images) was added to each channel to facilitate thresholding. Collected images were brightness- and contrast-adjusted and analyzed with Fiji.^64^

### FRAP Measurements

The FRAP measurements were performed on a Zeiss LSM 800 confocal microscope equipped with a 63× (NA = 1.4) Plan-Apochromat oil objective (Carl Zeiss AG, Germany). The dsGUVs were sealed within a BSA-coated observation chamber and placed in a thermostatic chamber at 25 °C. Two circular areas of 2.5 μm radius were defined as the probed area at the bottom of each dsGUVs determinated by *z*-stack profiling beforehand: (1) as bleaching spot and (2) as reference spot (unbleached) for data correction. Using bleaching, experimental regions, and time series options in Zeiss Zen software (Zen v2.3), 10 images (laser power, 1.0%) were recorded prior to bleaching (100 iteration; laser intensity, 100%) and 100 images after bleaching (laser intensity, 1.0%) as depicted in Figure 1B. A 561 nm laser (excitation of Liss Rhod B) was used for the FRAP measurements, with a pinhole aperture set to one Airy Unit. The 247 × 247 pixels images were recorded with an integration of 71.85 ms per image. The diffusion coefficient was extracted from the acquired images using an adapted MATLAB (MathWorks, Inc.) code as described previously,^3^ where a nonlinear least-square fit was applied to the normalized fluorescence intensity from the recovery phase. Details are presented in the Supporting Information note 4.

### Zeta Potential Measurements

The zeta potentials of SUVs and free-standing GUVs were diluted to 50 μM PBS at the desired pH. Measurements were performed on a Malvern ZetaSizer Nano ZS in a folded capillary zeta cell (Malvern). The refractive index of the dispersant was set to 1.330 and the viscosity to 0.882 cP with a dielectric constant of 79. The κ·α value was set to 1.5. The refractive index on the colloids was set to 1.42, which matched the refractive index of the GUVs. A 5 min equilibration time was used prior to every measurement for thermal stabilization. For each experimental condition, samples were measured in triplicate, with a minimum of 10 runs per measurements. The maximal voltage was set to 25 V to minimize potential oxidation/reduction effect of the lipid with the capillary electrodes.

### FRET Assay

Lipid mixing between the pH-sensitive/positively charged liposomes and negatively charged liposomes acting as compartment was investigated by FRET between donor and acceptor dyes as a function of pH. The FRET probe NBD-PE and Liss Rhod B PE, acting as donor and acceptor, respectively, were formulated within the same pH-sensitive/cationic liposomes resulting in a quenching of the NBD signal due to FRET toward Rhod B PE lipids located in the close vicinity. Upon lipid mixing with the negatively charged liposomes, the mean distance between the NBD and Rhod B lipids increases, resulting in an a rapid unquenching of the NBD fluorescence. The pH-sensitive SUVs were composed of DOBAQ/DOPG/DOPC/NBD-PE/Liss Rhod B PE (60/20/18/1/1 mol %), cationic liposomes were composed of DOTAP/DOPC/NBD-PE/Liss Rhod B PE (30/68/1/1 mol %), while anionic liposomes were formulated with DOPC/DOPE/DOPG/Cholesterol (45/20/20/15 mol %). All liposomes were prepared at a final lipid concentration of 10 mM by thin-film rehydration. Lipids films were rehydrated with 10 mM Tris-HCl, 50 mM NaCl at pH 8.5, swelled for 20 min, vortexed for 30 s, and extruded 21 times 100 nm etch-polycarbonate membrane (Whatman) at room temperature with a mini extruder (Avanti Polar Lipids). To investigate the lipid mixing at various pH values, different buffers were prepared to cover a pH range from pH 3.0 to pH 8.5, with 0.5 pH unit increments, and adjusted with HCl/NaOH. All buffers included 50 mM NaCl and 10 mM of the buffering compound. For pH 3.0 to 5.5, we used sodium acetate; for pH 6.0 to 7.0, we used 2-(*N*-morpholino)ethanesulfonic acid (MES); and for pH 7.5 to 8.5, we used Tris-HCl. For lipid mixing experiments, 2 μL of donor liposomes (either pH-sensitive, or cationic liposomes) were added to 1998 μL of the corresponding buffer, and the baseline fluorescence was recorded (*F*_min_). Then, 495 μL of the resulting donor liposome solution was mixed with 5 μL of the negatively charged liposomes, mixed, and measured after 10 min of incubation at room temperature (*F*). Finally, 180 μL of the resulting solution was mixed with 20 μL of a 1 wt/wt % Triton-X100, gently mixed, and measured after 10 min (*F*_max_). The percentage (%) of lipid mixing was calculated by 100 × (*F* – *F*_min_)/(*F*_max_ – *F*_min_). Measurement were performed in triplicates with a Tecan Spark plate reader in Flat 96 wells plate OptiPlate Black (PerkinElmer), Ex/Em = 465/520 nm, number of flashes = 50, settle time = 150 ms.

### p*K*_a_ Evaluation by TNS Assay

To evaluate the p*K*_a_ of the different pH-sensitive lipids, a TNS-based assay was employed. Briefly, liposomes were formulated with pH-sensitive lipids/DOPC (30/70 mol %) in PBS pH 6.5 at a final lipid concentration of 6 mM. A 1 mM TNS solution in 9:1 (v/v) EtOH/Milli-Q water was prepared. Then, liposomes were diluted to 30 μM in the presence of a final TNS concentration of 10 μM in 200 μL per well of a 96-well plate with buffers containing 10 mM 4-(2-hydroxyethyl)-1-piperazineethanesulfonic acid, 10 mM 4-morpholineethanesulfonic acid (MES), 10 mM ammonium acetate, and 130 mM NaCl, where pH was adjusted in the range of 2.5 to 11 by 0.5 increments with HCl/NaOH. Afterward, the fluorescence of TNS was measured in triplicate at each pH using a Tecan Spark plate reader in a flat 96-well plate OptiPlate Black (PerkinElmer), Ex/Em = 321/431 nm, number of flashes = 30, settle time = 150 ms. Fluorescence of each liposome formulation at the various pH was then corrected and normalized according to the value at pH 2.5. A sigmoid function was applied with MATLAB (MathWorks) to the fluorescence data, and p*K*_a_ of the pH-sensitive lipid was approximated as the pH at the point of half maximal fluorescence intensity.

### Evaluation of the SUV-to-GUV Conversion Efficiency

pH-sensitive SUVs employed to assemble and produce free-standing GUVs through a pH trigger were diluted to 50 μL in buffer and mixed with 50 μL isopropanol and vortexed to generate standards (2, 5, 15, 50, and 100 μM standards final concentration), as presented in Figure 4A. Then, 10 μL of free-standing GUVs (released in a total aqueous phase of 100 μL final) was diluted to 50 μL in buffer and mixed with 50 μL isopropanol and vortexed. Fluorescence intensity of the Liss Rhod B-labeled DOPE lipids was assessed using a Tecan Spark plate reader in a flat 96-well plate OptiPlate Black (PerkinElmer), Ex/Em = 535/595 nm, number of flashes = 50, settle time = 150 ms, volume per well = 100 μL. Samples and standards were measured in triplicate. The SUV-to-GUV conversion efficiency (%) was evaluated by the following equation
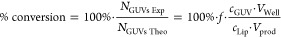
where *f* is a dilution factor, *c*_GUV_ is the lipid concentration of the free-standing GUVs measured by the calibration curve generated with the precursor SUVs, *V*_well_ is the well’s volume used to assess the fluorescence intensity by a plate reader measurement, and *V*_Prod_ is the total volume of aqueous phase used to generate the W/O emulsion. Further details on calculation and generation of the presented equation are provided in the Supporting Information note 5.

### Partitioning Assay

The Krytox contamination in fluorosurfactant was assessed by a partitioning assay. In the first steps, 100 μL of Krytox standards in HFE-7500 (0–1 mM Krytox, 0.1 mM steps) and fluorosurfactant samples [1.4 wt/wt % for commercially available PEG-based fluorosurfactant (CFS) or 2.5 mM for the self-synthetized PEG-based fluorosurfactant (SynFS)] in HFE-7500 were prepared. Then, 100 μL of 1 mM rhodamine 6G solution in liquid chromatography–mass spectrometry grade water was layered onto the fluorinated phase. Careful pipetting ensures that no emulsion was generated through the process. The mixtures of the two immiscible liquid were incubated for at least 48 h and protected from light. Afterward, 10 μL of the HFE-7500 phases were collected and diluted to 100 μL with HFE-7500. The partition was calculated by measuring the change in absorbance at 530 nm with a Tecan Spark plate reader in a flat 96-well plate (TPP Techno Plastic Product) as a function of Krytox concentration, as depicted in Figure 4A. The calibration curve was employed to evaluate the Krytox impurity. The molar percentage (mol %) of Krytox impurity was defined as the ratio of Krytox concentration divided by the correct concentration of PEG-based fluorosurfactants, which excluded the mass of Krytox impurity.

### Data Analysis

Numerical data were analyzed and plotted with various self-written codes in MATLAB (2019a, MathWork). Data fitting was performed in with the curve fitting tool box (Mathwork). In all cases, a robust Bisquare regression method was applied. Fluorescence images were analyzed with Fiji. For the pH measurement inside W/O droplets, droplet detection, localization, and assessment of their fluorescence intensity were achieved through a self-written macro in Fiji. Statistical analysis was performed with Prism 9 (GraphPad Software). An unpaired *t*-test analysis, while assuming a Gaussian distribution presenting similar standard deviation (parametric without correction), was used to determine the statistical significance of the pH- and Mg^2+^-mediated method of assembly (Figure 4). Alternatively, a two-way analysis of variance (ANOVA), with a Sidak’s multiple comparisons test, was used to determine the statistical significance of the pH- and Mg^2+^-mediated method of assembly as a function of the PEG-based fluorosurfactant (Figure S15). The sample size for figures requiring statistical analysis is stated within the corresponding figure caption.

## Supporting Information Available

General workflow of compartmentalized GUVs; summary of the various conditions for pH-mediated assembly of dsGUVs; TNS assay of DOBAQ lipid; ζ-potential measurements of pH-sensitive SUVs, negatively charged inner compartment (none pH-sensitive) and released GUVs at different pH values; assembly of compartmentalized dsGUVs with citrate buffers; multicompartment assembly of dsGUVs with DOTAP lipids; lipid mixing assay of DOTAP; microfluidics assembly of dsGUVs with citrate buffer; size distribution measurements of between methods of assembly; Krytox-mediated extraction of solute and Krytox contamination assay; dsGUVs assembly in 10 mM KH_2_PO_4_/K_2_HPO_4_ pH 7.4; TNS assay of DODMA lipid; assembly of multicompartment dsGUVs with DODMA; encapsulation of SUVs in W/O droplets in the absence of Mg^2+^; assessment of the SUVs-to-GUVs conversion efficiency by CLSM and fluorescence spectroscopy; calibration curve of SUVs in 1:1 isopropanol/buffer; CLSM of F-Actin encapsulation with and without a compartment in dsGUVs, and on glass slides; detailed buffer composition used in the various figures; impact of the PEG-based fluorosurfactant on the SUV to GUV conversion for pH- and Mg^2+^-mediated assembled dsGUVs; approximation of the time of diffusion of an SUV to the droplet periphery; FRAP analysis; and SUV-to-GUV conversion efficiency calculation (PDF)
pH-sensitive SUVs entrapped in W/O droplets in the presence of citrate buffer pH 5 using a two inlets microfluidics (AVI)
Mechanical splitter module with a single inlet for production of multicompartment GUVs by microfluidics (AVI)
F-actin cytoskeleton inside a free-standing GUV (AVI)

## Supplementary Material

sb1c00472_si_001.pdf

sb1c00472_si_002.avi

sb1c00472_si_003.avi

sb1c00472_si_004.avi
