# Supplementary material for: pH-Triggered Assembly of Endomembrane Multicompartments in Synthetic Cells
Source: ACS Synth Biol. 2021 Dec 10;11(1):366–82. doi: 10.1021/acssynbio.1c00472 (PMC8787813; doi:10.1021/acssynbio.1c00472)
Supplement: Supplementary file 1 — sb1c00472_si_001.pdf [file sb1c00472_si_001.pdf]

Supplementary information for: pH-Triggered Assembly of Endomembrane Multicompartments in Synthetic Cells

Félix Lussier[1,2], Martin Schröter[1,2], Nicolas J. Diercks[1,2], Kevin Jahnke[3,4], Cornelia Weber[1,2], Christoph Frey[1,2], Ilia Platzman*[1,2], Joachim P. Spatz*[1,2,5]*

[1]Department of Cellular Biophysics, Max Planck Institute for Medical Research, Jahnstraße 29, D-69120 Heidelberg, Germany

[2]Institute for Molecular Systems Engineering (IMSE), Heidelberg University, Im Neuenheimer Feld 225, D-69120 Heidelberg, Germany

[3]Max Planck Institute for Medical Research, Biophysical Engineering Group, Jahnstraße 29, D-69120 Heidelberg, Germany

[4]Department of Physics and Astronomy, Heidelberg University, D-69120 Heidelberg, Germany

[5]Max Planck School Matter to Life, Jahnstraße 29, D-69120 Heidelberg, Germany

Correspondence: Felix.lussier@mr.mpg.de; Ilia.Platzman@mr.mpg.de; Spatz@mr.mpg.de

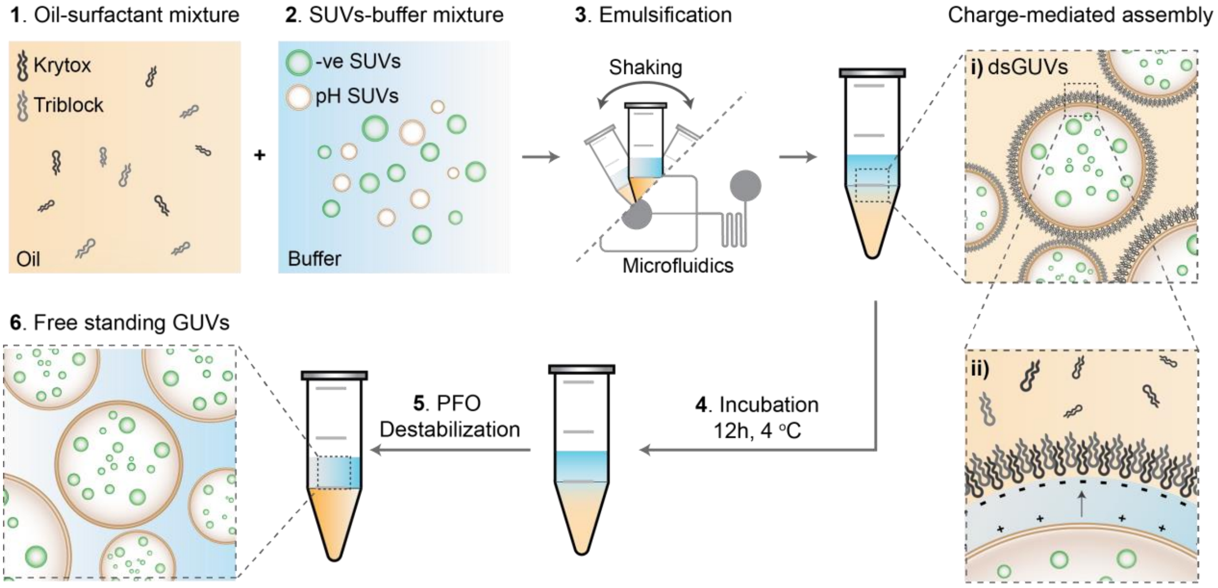

Figure S1: Schematic workflow for giant unilamellar vesicles assembly by a droplet-stabilized approach mediated by pH. First, an oil-surfactant mixture (**1**) which is composed of a PEG-based fluorosurfactant, PFPE carboxylic acid fluorosurfactant (i.e. Krytox) in fluorinated oil (HFE-7500) is mixted with a second mixture (**2**) composed of small unilamellar vesicles (SUVs) in the desired buffer. Here, one SUV population incorporate a pH sensitive lipid, while the other is permanently negatively charged. The two mixtures are then emulsified via either mechanical shaking (i.e. vortexing), or by droplet-based microfluidics (**3**). The resulting W/O emulsion (**i**) imply the recruitment of the various surfactant in the oil phase to the periphery of the droplets. The presence of Krytox generate a net negative charge at the interface. In absence of Krytox surfactant in the oil mixture, no charge mediated assembly/recruitment of the SUVs is observed. Based on the experimental conditions, the pH of the W/O droplet now acidic lead to the ionization of the pH sensitive lipids and trigger a selective charge-mediated recruitment of the pH sensitive SUVs to the droplet periphery while sequestrating the other negatively charged SUVs within the droplet lumen (**ii**). The resulting droplet-stabilized giant unilamellar vesicles (dsGUVs) are incubated for a period of 12h, at 4 °C (**4**) prior to the destabilization of the W/O emulsion via the addition of 1H,1H,2H,2H- perfluoro-octanol (PFO) to the droplet suspension (**5**). Once the emulsion if broken, the free standing GUVs are collected (**6**).

Table S1: Summary of conditions employed to adjust the intraluminal pH of W/O droplets.

pH adjustment	Final buffer(s) concentration and composition	Oil-surfactant mixture (in HFE-7500)	Bulk Assembly?	Microfluidics Assembly?
**Citrate buffer**	50 mM Citrate pH 5; 15 mM TrisHCl	1.4% wt/wt PEG-Based fluorosurfactant; 10 mM Krytox	Yes	Yes, but not with mechanical splitter
**acetic acid in HFE-7500**	50 mM KH2PO4/K2HPO4, 75 mM KCl	1.4% wt/wt PEG-Based fluorosurfactant; 10 mM Krytox; 36 mM acetic acid	Yes, direct or upon oil substitution	Yes, direct* or upon oil substitution
**Acidification only by Krytox**	10 mM KH2PO4/K2HPO4, 140 mM KCl	2.5 mM PEG-Based Fluorosurfactant; 10 mM Krytox	Yes	Yes

* 3% wt/wt PEG-Baed fluorosurfactant helps to minimized droplet coalescence when an acidic oil is directly employed for dsGUVs assembly.

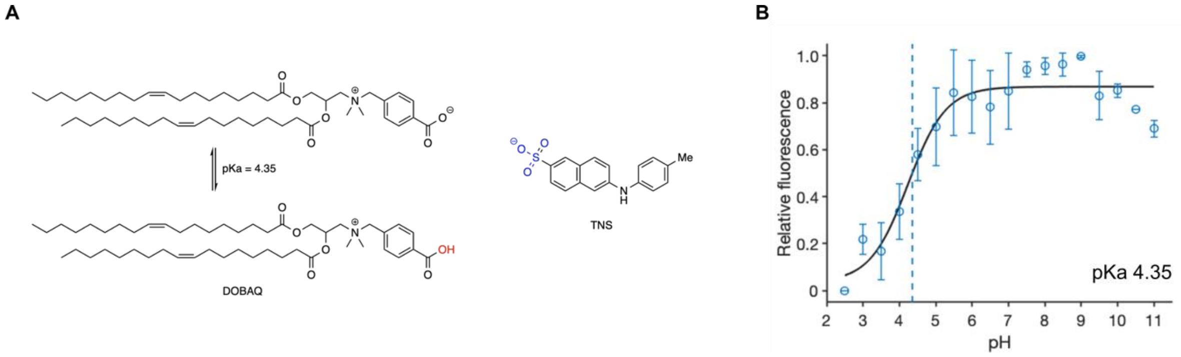

Figure S2: Evaluation of the pK_a of DOBAQ by TNS assay. (**A**) Molecular structure of DOBAQ and TNS. The carboxylic group in acidic pH, acting as a hydrogen bond donor is highlighted in red, while the sulfonate group of TNS, acting as a hydrogen bond acceptor is highlighted in blue. (**B**) TNS-based assessment of the pKa value of the pH sensitive SUVs. The pKa is defined as the half-maximum, corresponding to 50 percent of protonated DOBAQ lipid, extracted from a fitted sigmoid curve. The pKa is highlighted by the hatched vertical line. Mean ± S.D. are presented, $n = 3$.

Surprisingly, the TNS response showed a decrease in fluorescence intensity in acidic environment, even though an increase intensity was expected based on previously published experiments.[1-4] The molecular structure of the pH sensitive moieties was shown by Walsh and coworkers to impact its interaction with TNS, mitigating, to some extent, the expected rise in fluorescence from TNS.[5] Compared to most potent pH sensitive lipids, typically possessing tertiary amines[2, 6], DOBAQ possesses a permanent positively charged quaternary amine sterically hindered by a carboxylic acid function located at the forefront of the lipid headgroup (Fig. S1A). As a result, DOBAQ possesses a zwitterionic state in physiological condition, while acidic environment leads to the protonation of the carboxylic acid group rendering DOBAQ positively charged (Fig S1A). We hypothesize that the decrease in fluorescence is associated to the improved capability of DOBAQ to act as an hydrogen bond donor toward the negatively charged TNS at low pH, leading to an apparent increase of the polarity of the local lipophilic environment thus explaining the shape the of TNS assay (Fig S1B).

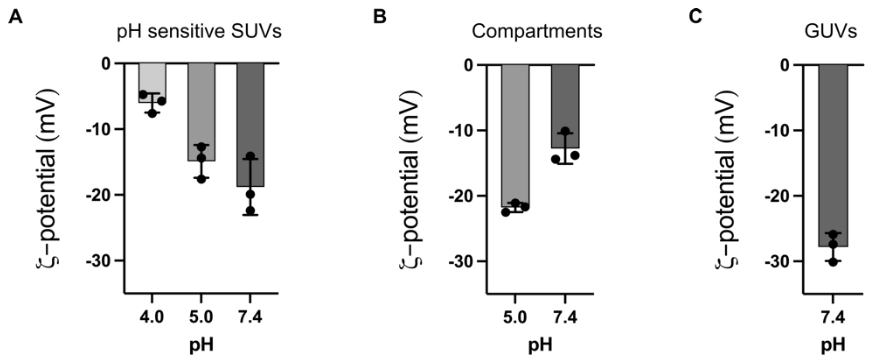

Figure S3: Evaluation of the charges of the two population of SUVs as a function of pH, and the resulting free-standing GUVs in physiological condition assembled by a pH trigger. (**A**) the pH sensitive SUVs containing DOBAQ/EggPC/EggPG/Liss Rhod B-labelled DOPE (60/19.5/20/0.5 mol%) and (**B**) the inner compartments SUVs containing EggPC/EggPG/ATTO488-labelled DOPE (79.5/20/0.5 mol%) by zeta-potential measurements. (**C**) Zeta-potential of the free-standing GUVs in physiological condition containing lipids originated from the pH sensitive SUVs in (**A**). In all cases, fluorescently-labelled lipids (Liss Rhod B, and ATTO488) were kept within the formulation to considered their potential effect of the charge of the colloids. Moreover, no interference with the instrument laser (He-Ne, 633 nm) is expected from to the presence of the fluorescent dyes whom possess an excitation wavelength of 561, and 488 nm for the Liss Rhod B and ATTO488, respectively. Mean ± S.D. (n = 3) are presented.

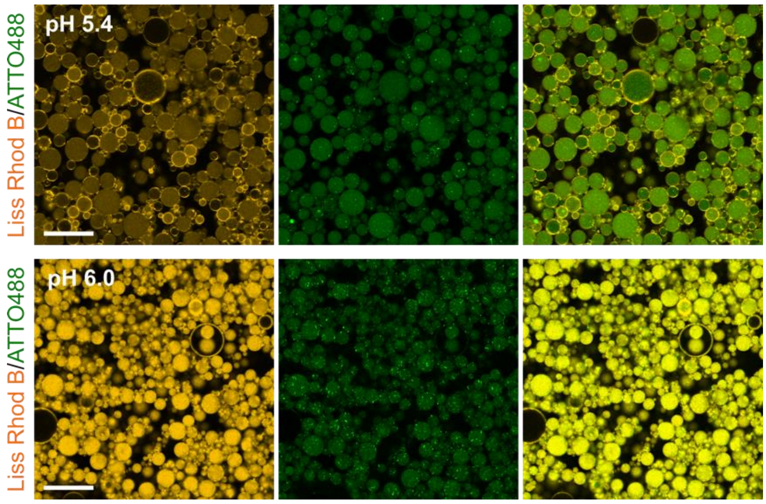

Figure S4: Assembly of droplet-stabilized GUVs via shaking in presence of 50 mM citrate buffer at various pH. By decreasing the intra luminal pH with a citrate buffer, pH sensitive SUVs exhibited a preferential fusion to the droplet periphery to generate a supported lipid bilayer. Scale bar, 50 µm. Oil phase: 2.5 mM PEG-based fluorosurfactant, 10 mM Krytox in HFE-7500. The pH sensitive SUVs composed of DOBAQ/eggPG/eggPC/Liss Rhod B-labelled DOPE (60/20/19.5/0.5 mol%) were co-encapsulated with negatively charged SUVs composed of DOPC/DOPG/ATTO488-labelled DOPE (79.5/20/0.5 mol%) within the W/O droplets stabilized by 1.4 wt/wt% fluorosrufactant and 10 mM Krytox. Various 50 mM citrate buffers we used to adjust the pH of the water phase. Scale bars: 50 µm. The acidification of the aqueous phase was achieved through the rapid addition of 100 mM citrate buffer at the desired pH to the aqueous phase right before emulsification. The final citrate concentration in the aqueous phase was 50 mM in all cases.

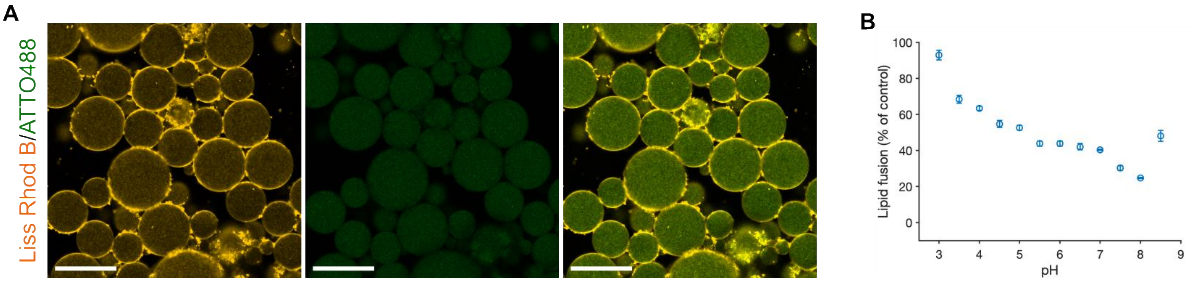

Figure S5: Assembly of multicompartment dsGUVs by mixing positively, and negatively charged SUVs population within water-in-oil droplets. (**A**) CLSM images of droplet-stabilized GUVs generated by encapsulating positively charged SUVs composed of DOTAP/DOPC/Liss Rhod B-labelled DOPE (30/69.5/0.5 mol%) and negatively charged SUVs, composed of DOPG/DOPC/ATTO488-labelled DOPE (30/69.5/0.5 mol%). Scale bars; 50 μm. (**B**) FRET assay measuring lipid mixing of positively charged SUVs with negatively charged SUVs. SUVs composed of DOTAP/DOPC/Liss Rhod B-labelled DOPE/DOPE-NBD (30/68/1/1 mol were mixed with unlabeled negatively vesicles at various pH. A relatively constant lipid mixing may be appreciated through, independent of the pH of the environment, which may account for the homogenous fluorescence signal within the dsGUVs lumen presented in (A). Mean ± S.D. are presented, $n = 3$.

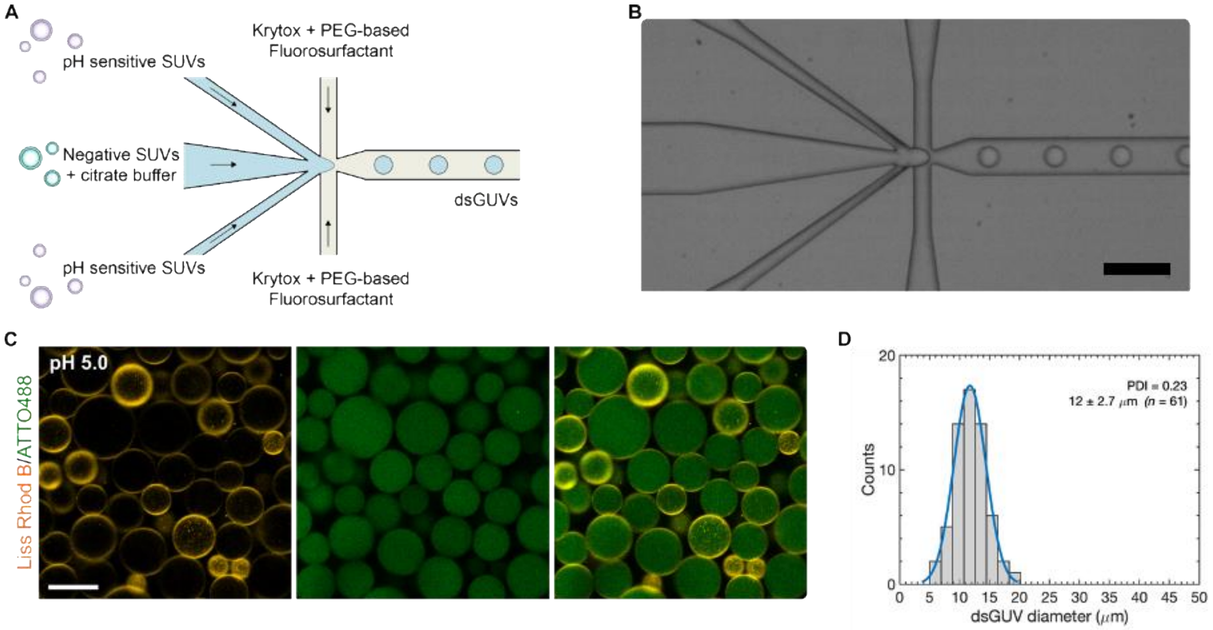

Figure S6: Generation of an endomembrane system within droplet-stabilized giant-unilamellar vesicles (dsGUVs) by microfluidics employing two aqueous inlets. (**A**) Schematic representation presenting the production of multicompartment dsGUVs trough the co-encapsulation of a pH sensitive SUVs containing DOBAQ lipids, and an inner compartment SUVs possessing a net negative charge in presence of a citrate buffer at pH 5. The droplets are stabilized through the use of a PEG-based fluorosurfactant and Krytox supplemented to the fluorinated oil-phase. (**B**) Optical micrograph of the microfluidic chip at the flow-focusing junction. Scale bar, 100 µm. (**C**) CLSM images of dsGUVs produced by microfluidics in presence of citrate buffer pH 5. The pH sensitive SUVs, composed of DOBAQ/EggPC/EggPG/Liss Rhod B-labelled DOPE (60/20/19,5/0,5 mol%) showed a preferential accumulation and fusion at the droplet periphery compared to the inner compartment composed of EggPC/EggPG/ATTO488-labelled DOPE (69,5/30/0,5 mol%). Scale bar, 20 µm. The two inlets device was applied to introduce independently: (1) 2 mM of the inner compartment with 100 mM citrate buffer pH 5 and (2) 3 mM of the pH sensitive SUVs. Due to the concomitant dilution of the two aqueous phase prior droplet encapsulation, the concentration was doubled compared to the bulk production achieved by emulsification by shaking. (**D**) Corresponding histogram of the size distribution of the droplets presented in (**C**). The blue lines correspond to the fitted Gaussian distribution. The size distribution was obtained with ImageJ using manual thresholding, and manual measurements of the droplets/dsGUVs diameters. Data were analyzed with MatLab in order extract the Gaussian distribution, and evaluate the polydispersity (PDI) of the produced dsGUVs. During the production, we noted a significant variation of the pressure in between the two inlets, which led to an increase polydispersity in droplet size. We further observed a potential clogging of the inlet channels, resulting from the rapid drop in pH promoting the aggregation of the pH sensitive SUVs.

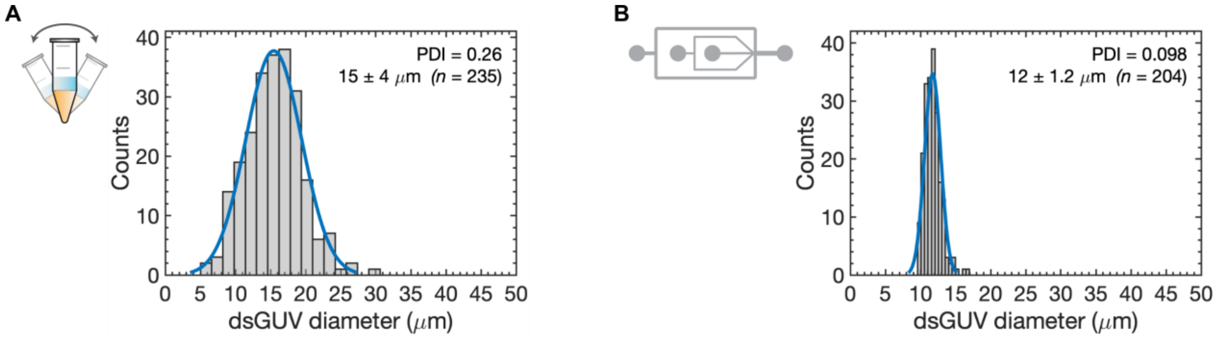

Figure S7: Size distribution of droplet-stabilized GUVs assembly by shaking (**A**) or microfluidic (**B**) employing two aqueous inlets (see Fig. S6A-B). Acidification of the aqueous phase was controlled by the use of citrate buffer pH 5. The size distribution was obtained with ImageJ using manual thresholding, and manual measurements of the droplets/dsGUVs diameters. Data were analyzed with MatLab in order extract the Gaussian distribution, and evaluate the polydispersity (PDI) of the produced dsGUVs.

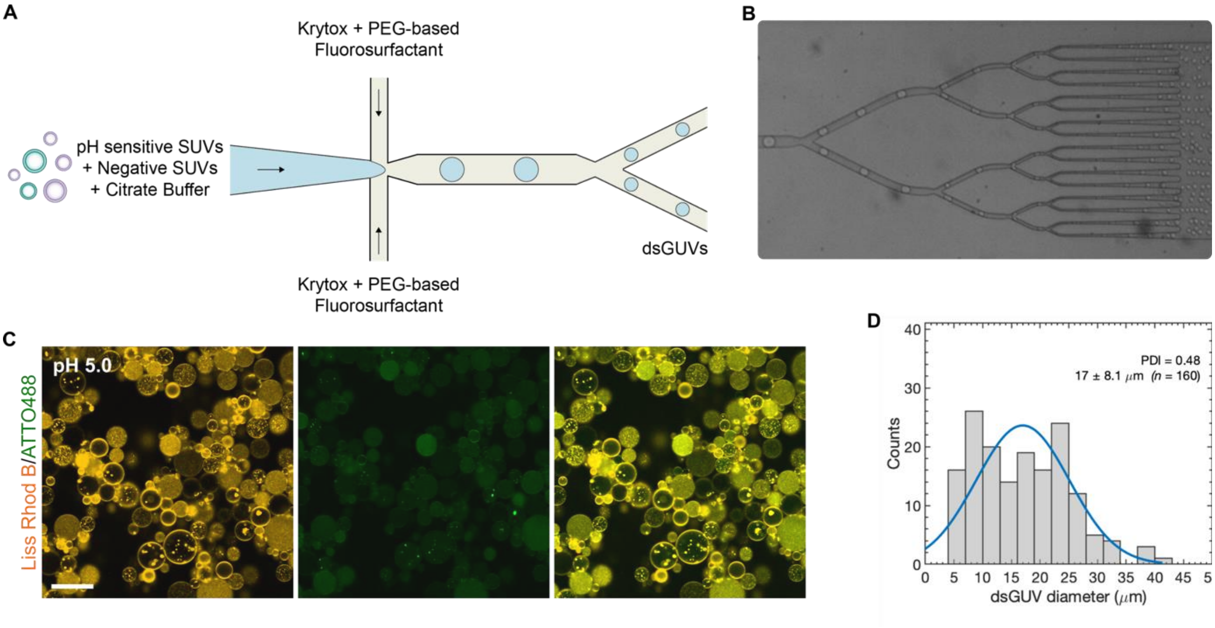

Figure S8: Attempt to generate an endomembrane system within small droplet-stabilized giant-unilamellar vesicles (dsGUVs) by microfluidics employing mechanical splitters. (**A**) Scheme presenting the production of multicompartment dsGUVs trough the co-encapsulation of a pH sensitive SUVs containing DOBAQ lipids, and a inner compartment SUVs possessing a net negative charge in presence of a citrate buffer at pH 5. The droplets are stabilized through the use of a PEG-based fluorosurfactant and Krytox supplemented to the fluorinated oil-phase. (**B**) Optical micrograph of the microfluidic chip at the sequential mechanical splitting units exhibiting a concomitant reduction in channel width. (**C**) CLSM images of dsGUVs produced by mechanical splitter in presence of citrate buffer pH 5. The pH sensitive SUVs, composed of DOBAQ/EggPC/EggPG/Liss Rhod B-labelled DOPE (60/20/19,5/0,5 mol%) showed no preferential recruitment and fusion at the droplet periphery compared to the inner compartments composed of EggPC/EggPG/ATTO488-labelled DOPE (69,5/30/0,5 mol%), while also presenting numerous aggregated SUVs. Scale bar, 50 µm. The two inlets, not schematized in (**A**) due to the presence of a 500 µm length channel post inlets but prior the flow-focusing junction, enabled the independent introduction of: (1) 2 mM of the inner compartment with 100 mM citrate buffer pH 5 and (2) 3 mM of the pH sensitive SUVs. Due to the concomitant dilution of the two aqueous phase prior droplet encapsulation, the concentration was doubled compared to the bulk production achieved by emulsification by shaking. (**D**) Corresponding histogram of the size distribution of the droplets presented in (**C**). The blue lines correspond to the fitted Gaussian distribution. The size distribution was obtained with ImageJ using manual thresholding, and manual measurements of the droplets/dsGUVs diameters. Data were analyzed with MatLab in order extract the Gaussian distribution, and evaluate the polydispersity (PDI) of the produced dsGUVs. During the production, we noted a significant variation of the pressure in between the two inlets, which led to an increase polydispersity in droplet size. We further observed a potential clogging of the inlet channels, resulting from the rapid drop in pH promoting the aggregation of the pH sensitive SUVs. Data showed the large polydispersity associated to the aggregation of the SUVs at low pH and the concomitant clogging of the microfluidics channels.

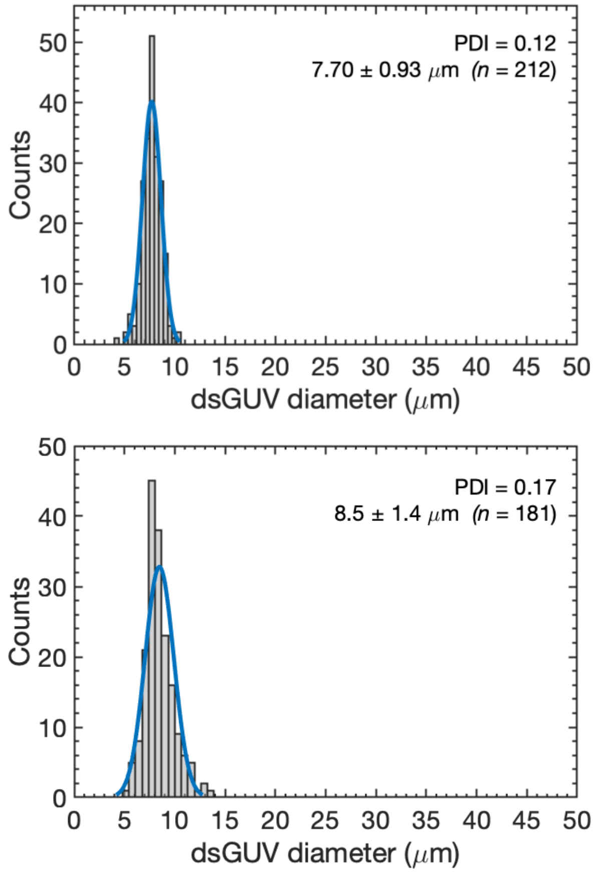

Figure S9: Histogram of the size distribution of the droplets (Top), and droplets-stabilized giant unilamellar vesicles possessing an endomembrane system (Bottom) measured by CLSM of the image presented in Fig.2D produced by microfluidic mechanical splitters. Herein, droplets encapsulating various SUVs were produced in absence of supplemented acid, while the dsGUVs assembly was triggered upon exposing the droplets to an acid-containing oil-surfactant phase. The blue lines correspond to the fitted Gaussian distribution. The size distribution was obtained with ImageJ using manual thresholding, and manual measurements of the droplets/dsGUVs diameters. Data were analyzed with MatLab in order extract the Gaussian distribution, and evaluate the polydispersity (PDI) of the dsGUVs.

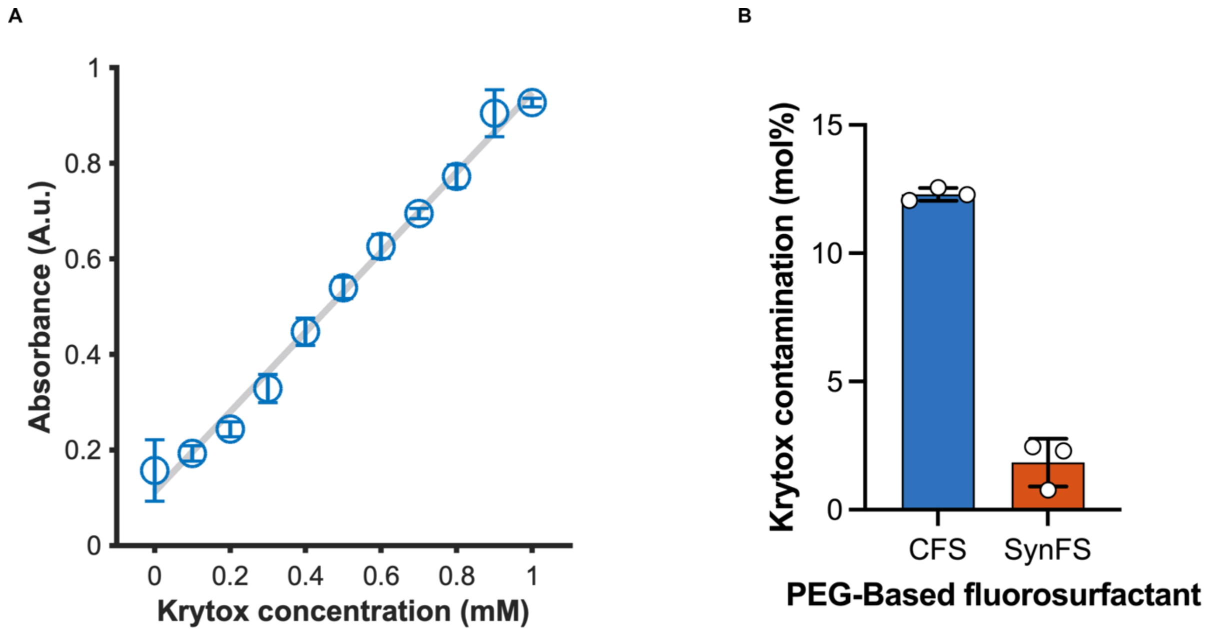

Figure S10: Krytox-mediated extraction of solutes. (**A**) Calibration curve of rhodamine 6G measured in the fluorous phase (HFE-7500) by absorbance spectroscopy at 530 nm (R^2 = 0.9912) at various concentration of Krytox supplemented within the fluorous phase. Mean ± S.D. (*n* = *3*) are presented. (**B**) Evaluation of the Krytox contamination within PEG-based fluorosurfactants by macroscopic partitioning Experiment. Aqueous solution of rhodamine 6G (1 mM) are exposed to the fluorous phase supplemented with different PEG-based fluorosurfactants: Commercially available PEG-based fluorosurfactant (CFS, 1.4 wt/wt% ≈ 3.8 mM) from Ran Technology, or a self-synthetized PEG-based fluorosurfactant (SynFS, 2.5 mM) for the present study. The rhodamine 6G signal was assessed into the fluorous phase by absorbance at 530 nm, and directly correlated to the Krytox impurity in the PEG-based fluorosurfactant due to a stochiometric association from (**A**).[7, 8] Mean ± S.D. (*n* = *3*) are presented.

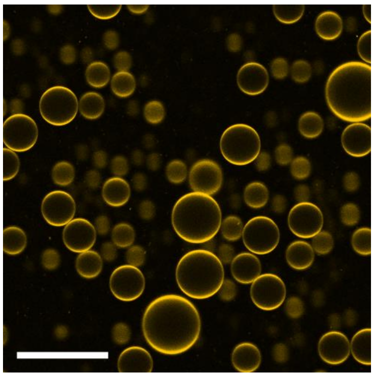

Figure S11: pH mediated assembly of droplet stabilized giant unilameller vesicles (dsGUVs). The aqueous phase was composed of 1.5 mM pH sensitive SUVs composed of DOBAQ/DOPG/DOPE/DOPC/Liss Rhod B labelled-DOPE (30/20/10/39.5/0.5 mol%) in 10 mM KH_2PO_4/K_2HPO_4 pH 7.4. The oil-surfactant mixture was composed of 2.5 mM PEG-based fluorosurfactant, and 10 mM Krytox in HFE-7500. The dsGUVs were produced by the one-pot shaking method, with an aqueous:oil ratio of 1:2 (50 : 100 µL). Scale bar: 50 µm.

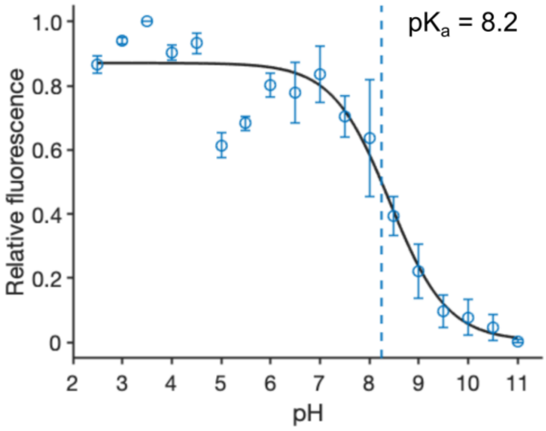

Figure S12: TNS assay to assess the apparent pKa of the pH sensitive lipid DODMA incorporated in SUVs composed of DODMA/DOPC (30/70 mol%). The increase in TNS signal correlated to the protonation of the amine functional group leading to an increase electrostatic attraction with TNS leading to an unquenching of its fluorescence. The pKa is defined as the half-maximum, corresponding to 50 percent of protonated DODMA lipid, extracted from a fitted sigmoid curve. The pKa is highlighted by the hatched vertical line. Mean ± S.D. are presented, $n = 3$.

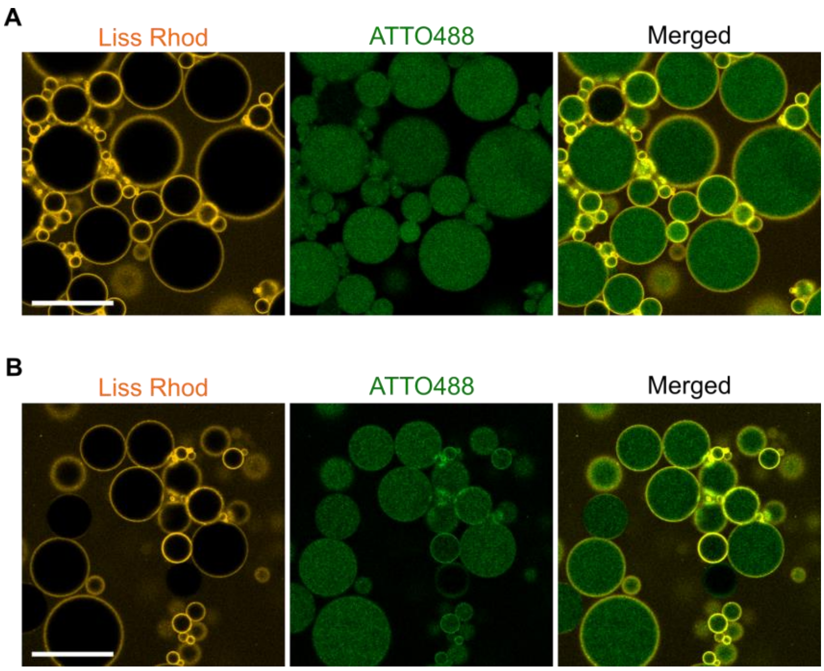

Figure S13: Self-assembly of multicompartment dsGUVs by Krytox acidification of the droplet lumen through co-encapsulation of two SUVs population. (**A**) Encapsulation of negatively charged SUVs composed of DOPG/DOPC/ATTO488-labelled DOPE (30/69.5/0.5 mol%) in 10 mM KH_2PO_4/K_2HPO_4, 140 mM KCl, pH of 7.4. (**B**) Encapsulation of neutral SUVs composed of DOPC/ATTO488-labelled DOPE (99.5/0.5 mol%) in 10 mM KH_2PO_4/K_2HPO_4, 140 mM KCl, pH of 7.4. In both cases, 1.5 mM of the pH sensitive SUVs composed of DODMA/DOPG/DOPC/DMG-PEG/Liss Rhod B-labelled DOPE (30/15/50.5/4/0.5 mol%) in 10 mM KH_2PO_4/K_2HPO_4, 140 mM KCl, pH of 7.4. The oil phase was composed of 2.5 mM PEG-based fluorosurfactant supplemented with 7.5 mM Krytox in HFE-7500.

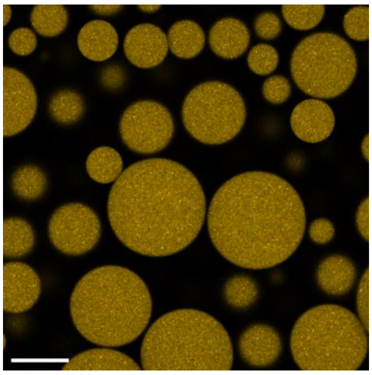

Figure S14: Production of dsGUVs in absence of Mg^{2+} ions. Herein, 1.5 mM SUVs containing DOBAQ/DOPG/DOPC/DOPE/Liss Rhod B-labelled DOPE (30/20/39.5/10/0.5 mol%) in 30 mM Tris buffer pH 7.4 were encapsulated W/O droplets stabilized by 2.5 mM PEG-based fluorosurfactant and 10 mM Krytox in HFE-7500. The homogeneous fluorescence signal in the droplets' lumen highlight that no pH-mediated assembly is expected in these experimental conditions. Scale bar: 50 µm

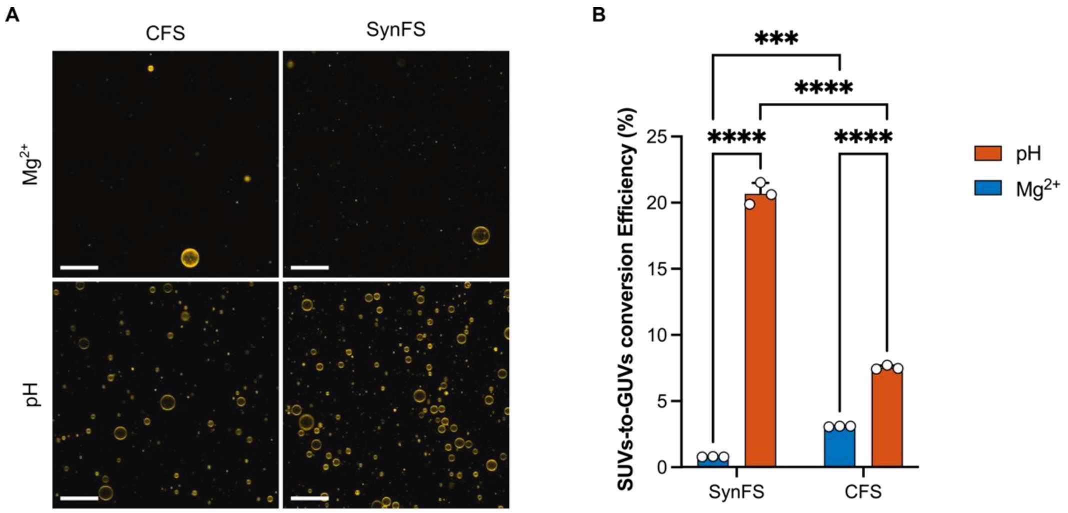

Figure S15: (**A**) CLSM images of the GUVs produced by pH- or Mg^{2+}-mediated assembly with the use of various PEG-based fluorosurfactant. In all cases, 1.5 mM of pH sensitive SUVs containing DOBAQ/DOPG/DOPC/DOPE/Liss Rhod B-labelled DOPE (30/20/39.5/10/0.5 mol%) were encapsulated into W/O droplets stabilized by the corresponding PEG-based fluorosurfactant. In the case of Mg^{2+}-mediated assembly, the aqueous phase was supplemented with 10 mM $MgCl_2$ and 30 mM Tris buffer pH 7.4. In the case of the pH-mediated assembly, the aqueous phase was 10 mM KH_2PO_4/K_2HPO_4, 140 mM KCl, pH 7.4. For the commercially available PEG-based fluorosurfactant (CFS), the oil-phase contained 1.4 wt/wt% CFS, and 10 mM Krytox in HFE-7500. In the case of SynFS, the oil-phase contained 2.5 mM SynFS, and 10 mM Krytox in HFE-7500. CLSM for the SynFS are presented in Fig. 4A, and included herein for visual comparison. (**B**) Two-way analysis of variance (ANOVA) of the SUVs-to-GUVs conversion efficiency as a function of the method of assembly, and the choice of the PEG-based fluorosurfactant assessed by fluorescence spectroscopy. Data for SynFS are presented in Fig 4B, but were included herein for the two-way ANOVA, and visual comparison. Mean ± S.D. (n = 3) are presented. ***P<0.001; ****P<0.0001.

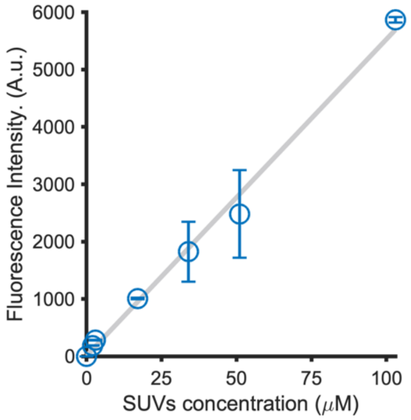

Figure S16: Calibration curves of SUVs incorporation Liss Rhod B labelled-DOPE lipids in a 1:1 isopropanol:buffer mixture. The buffer corresponded to 10 mM KH_2PO_4/K_2HPO_4, 140 mM KCl at pH 7.4. Mean ± S.D. are presented (n = 3), R^2 = 0.9912.

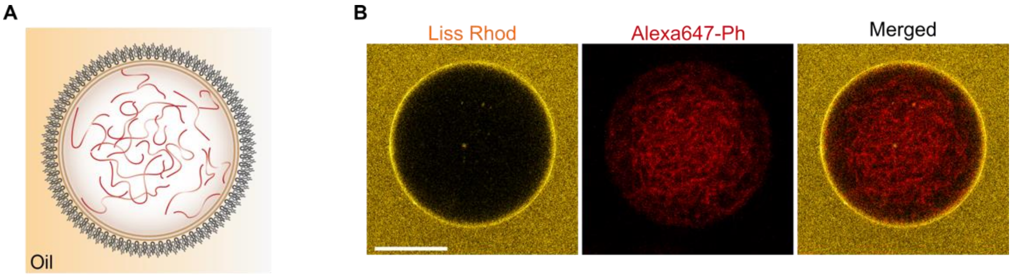

Figure S17: Reconstruction of an F-actin cytoskeleton within a synthetic cell by pH mediated assembly of droplet-stabilized GUV. (**A**) Scheme presenting the encapsulation of F-actin and pH sensitive SUVs within W/O droplets stabilized by PEG-based fluorosurfactant and Krytox. Herein, the pH sensitive SUVs fused to the droplet periphery to entrap the F-actin upon acidification of the droplet lumen by Krytox. The resulting synthetic cells were released in physiological condition leading to the production of minimal synthetic cell possessing an F-actin cytoskeleton as presented in figure 5B. (**B**) CLSM of droplet-stabilized GUVs encapsulating 1.5 mM pH sensitive SUVs composed of DOBAQ/DOPG/DOPE/DOPC/DMG-PEG/Liss Rhod B- labelled DOPE (30/20/10/35.5/4/0.5 mol%) and 5 µM of F-actin labelled with Phalloidin-Alexa647. Scale bar: 10 µm.

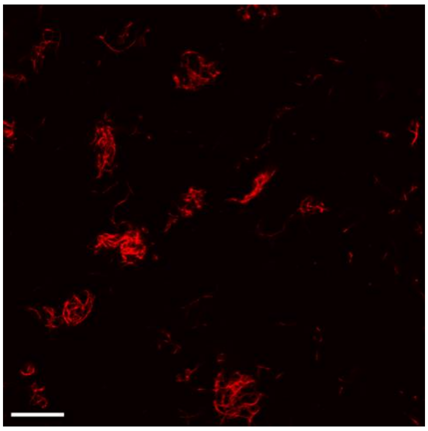

Figure S18: CLSM image of F-actin-Phalloidin-647 bundles and filaments adsorbed on the BSA-coated observation chamber. The F-actin located in the buffer following the release of the F-actin-loaded dsGUVs in physiological conditions originate from rupture of GUVs during the release process which liberate the entrapped F-actin into the buffer. Scale bar: 50 µm.

A

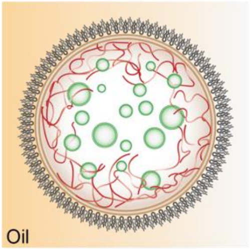

B

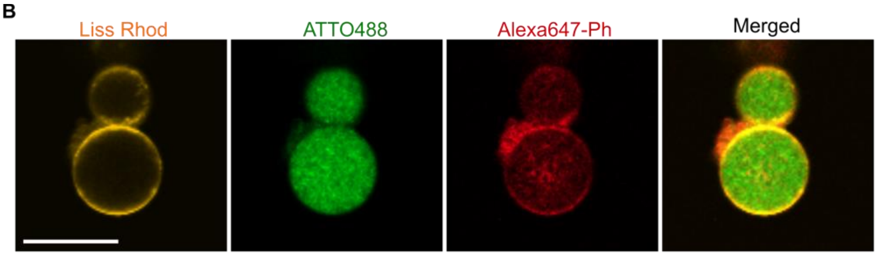

Figure S19: Reconstruction of an F-actin cytoskeleton and an endomembrane within a synthetic cell by pH mediated assembly of droplet-stabilized GUV. (**A**) Scheme presenting the encapsulation of both F-actin and negatively charged compartments along with pH sensitive SUVs within W/O droplets stabilized by PEG-based fluorosurfactant and Krytox. The pH sensitive SUVs fused to the droplet periphery to entrap the F-actin upon acidification of the droplet lumen by Krytox. The resulting synthetic cells were released in physiological condition leading to the production of minimal synthetic cell possessing both a F-actin cytoskeleton and and endomembrane system as presented in figure 5D. (**B**) CLSM of droplet-stabilized GUVs encapsulating 1.5 mM pH sensitive SUVs composed of DOBAQ/DOPG/DOPE/DOPC/DMG-PEG/Liss Rhod B-labelled DOPE (30/20/10/35.5/4/0.5 mol%), 1 mM of negatively charged SUVs composed of DOPG/DOPC/ATTO488-labelled DOPE (30/69.5/0.5 mol), and 5 µM of F-actin labelled with Phalloidin-Alexa647. Scale bar: 25 µm.

Supplementary note 1: Aqueous and oil-mixture composition use for the various method of assembly of GUVs by a pH trigger presented in the figures of the main text:

In all cases, a ratio of 1:2 water: oil was respected, with the typical working volume of 50 µL of aqueous:100 µL oil phase. All aqueous solutions were prepared in Milli-Q water, and all oil-surfactant mixture were prepared in HFE-7500. A 30 mM stock Krytox solution was prepared in HFE-7500.

Self-assembly of compartmentalized GUVs in presence of low pH citrate buffer: In all cases, the oil-surfactant mixture was composed of 1.4% w/w PEG-based fluorosurfactant, and 10 mM Krytox. **Fig. 1A**: 50 mM citrate buffer pH 5, 1.5 mM pH-sensitive SUVs (6 mM stock in 30 mM TrisHCl pH 7.4). **Fig 1C**: 50 mM citrate buffer pH 5, 1.5 mM pH-sensitive SUVs (6 mM stock in 30 mM TrisHCl pH 7.4), 1 mM negative SUVs (6 mM stock in 30 mM TrisHCl pH 7.4).

Self-assembly of compartmentalized GUVs with acidified oil-surfactant mixture – well-buffered aqueous phase: In all cases, except when explicitly mentioned, the aqueous phase was composed of 1.5 mM of pH-sensitive SUVs in 50 mM KH_2PO_4/K_2HPO_4 pH 7.4 and 75 mM KCl; **Fig. 2A**: 1.4% wt/wt PEG-based fluorosurfactant, 10 mM Krytox, and 4 (8, 18, or 36) mM acetic acid; **Fig 2B**: (**i**) Droplets were initially produced with 1.4% w/w PEG-based fluorosurfactant, and 10 mM Krytox. (**ii**) Excess of oil, located at the bottom of the Eppendorf due to higher density is removed, and 100 µL was added of an acidic oil composed of 1.4% wt/wt PEG-based fluorosurfactant, 10 mM Krytox, and 36 mM acetic acid; **Fig. 2C**: (**i**)1.4% w/w PEG-based fluorosurfactant, and 10 mM Krytox. (**ii**) Excess of oil was removed, and ~100 µL (roughly the amount removed) was added of an acidic oil composed of 1.4% wt/wt PEG-based fluorosurfactant, 10 mM Krytox, and 36 mM acetic acid. (**iii**) 3.0 % wt/wt PEG-based fluorosurfactant, 10 mM Krytox, and 36 mM acetic acid; **Fig 2D**: (**i**) The aqueous phase was composed of 1.5 mM of pH-sensitive SUVs in 50 mM KH_2PO_4/K_2HPO_4 pH 7.4 and 75 mM KCl, 1 mM of negatively charged SUVs in 50 mM KH_2PO_4/K_2HPO_4 pH 7.4 and 75 mM KCl; Oil-surfactant mixture: 1.4% w/w PEG-based fluorosurfactant, and 10 mM Krytox. (**ii**) Excess of oil was removed, and ~100 µL was added of an acidic oil composed of 1.4% wt/wt PEG-based fluorosurfactant, 10 mM Krytox, and 36 mM acetic acid.

Self-assembly of compartmentalized GUVs only mediated by acidification from Krytox – lower buffering capacity: In all cases, except when stated, DOBAQ was used as pH sensitive lipid in the corresponding formulation; **Fig 3E**: Aqueous phase: 1.5 mM pH sensitive SUVs (DODMA lipids), 1 mM of negatively charges SUVs (each populations) in 10 mM KH_2PO_4/K_2HPO_4 pH 7.4 and 140 mM KCl. Oil-surfactant mixture: 2.5 mM PEG-based fluorosurfactant, and 7.5 mM Krytox; **Fig. 4A**: (**Mg^{2+}**) Aqueous: 1.5 mM pH sensitive SUVs in 30 mM TrisHCl pH 7.4, 10 mM $MgCl_2$. Oil-surfactant mixture: 2.5 mM PEG-based fluorosurfactant, and 10 mM Krytox. (**pH**) Aqueous: 1.5 mM pH sensitive SUVs in 10 mM

KH_2PO_4/K_2HPO_4 pH 7.4 and 140 mM KCl. Oil-surfactant mixture: 2.5 mM PEG-based fluorosurfactant, and 10 mM Krytox; **Fig 4E**: Aqueous: 1.5 mM pH sensitive SUVs and 1 mM Calcein-loaded QDOPE SUVs in 10 mM KH_2PO_4/K_2HPO_4 pH 7.4 and 140 mM KCl. Oil-surfactant mixture: 2.5 mM PEG-based fluorosurfactant, and 10 mM Krytox.

Supplementary note 2: Impact of the PEG-based fluorosurfactant on the SUVs to GUVs conversion for pH- and Mg^{2+}-mediated assembled dsGUVs.

To maximize the production of GUVs, the Krytox concentration within the oil-surfactant mix needs to be finely tuned, especially in the case of Mg^{2+}-mediated assembly of dsGUVs. Commercial PEG-based fluorosurfactants (CFSs) possess a none negligible contamination by Krytox,[8] which is the common source of PFPE monomer for the di- and triblock fluorosurfactant synthesis.[9-12] Also, the composition of CFSs correspond to an *a priori* variable mixture of di- and triblock copolymer revealed by MALDI-TOF analysis by Haller and collegues.[8] This potential variability results in frequent re-optimization of the experimental conditions to maintain an efficient GUV production. To overcome these variabilities and evaluate the impact of the PEG-based fluorosurfactant on the SUVs to GUVs conversion efficiency, we synthetized a PEG-based fluorosurfactant (SynFS) possessing minimal Krytox contamination, as showed by the partitioning assay (Fig S8). We applied click chemistry to generate the PEG-based fluorosurfactant, composed of $PFPE_{7000}$-PEG_{1500}-$PFPE_{7000}$. The click-based coupling was favored over classical amide bond formation due to its improved control, reproducibility of the synthesis[13] compared to traditional synthetic routes[9, 12, 14] and ease of purification steps, which minimized the potential Krytox contamination.

In the case of the pH-mediated assembly of GUVs, we observed a further improvement in both the absolute number of GUVs (Fig. S12A) and the SUVs to GUVs conversion efficiency (Fig. S12B) compared to Mg^{2+} when the SynFS was used, but also compared to CFS. Interestingly, we noted that the conversion efficiency was reduced for Mg^{2+} when the SynFS was used instead of the CFS. These results further highlight the necessity to optimize the experimental conditions for maximal release efficiency, and that *a priori* other interactions associated to the molecular structure of the surfactants would need to be accounted for. Nevertheless, the joint usage of SynFS and pH showed a significant improvement in SUVs to GUVs conversion efficiency to empower the high throughput production of multicompartment synthetic cells.

Supplementary note 3: Approximation of the time of diffusion of an SUV to the droplet periphery[15]

By assuming 3D Brownian motion, the mean square displacement, $\langle r^2(t) \rangle$, of a spherical particle correspond to

$$\langle r^2(t) \rangle = 6D\langle t \rangle \quad (1)$$

Where D correspond to the diffusion coefficient of the particle. Thus, the average time $\langle t \rangle$ required for a particle to travel a distance of $\langle r^2(t) \rangle^{1/2}$ is

$$\langle t \rangle = \frac{\langle r^2(t) \rangle}{6D} \quad (2)$$

By applying the Stoke-Einstein equation of diffusion

$$D = \frac{k_B T}{6\pi\eta R} \quad (3)$$

Where k_B is the constant of Boltzmann, T the temperature, η the viscosity of the solution and R to the radius the particle in solution. Therefore, the average time $\langle t \rangle$ may be expressed as

$$\langle t \rangle = \frac{\langle r^2(t) \rangle}{6D} \times \frac{6\pi\eta R}{k_B T} = \frac{\langle r^2(t) \rangle \cdot \pi\eta R}{D k_B T} \quad (4)$$

By assuming a typical viscosity of 0.904 x 10^{-3} Pa · s for a phosphate buffer saline (PBS), a temperature of 298 K, and particle radius of 50 nm, the time require by a SUVs travelling from the center of the W/O droplet possessing a mean diameter of 15 µm to reach the periphery ($\langle r^2(t) \rangle^{1/2}$ = 7.5 µm; $\langle r^2(t) \rangle$ = 56.25 μm^2) will be roughly 400 milliseconds.

Alternatively, an SUV of 100 nm diameter would have a D of 24.13 μm^2 s approximated by the Stoke-Einstein equation (3).

Supplementary note 4: FRAP analysis

For the FRAP analysis, the normalized fluorescence intensity values were calculated as fellow:

$$I_{Normalized} = \frac{I_{Bleached} - I_{Reference}}{I_{Pre\ Bleached} - I_{Pre\ Reference}} \quad (5)$$

Where $I_{Bleached}$ correspond to the fluorescence intensity of the bleached spot, $I_{reference}$ is the fluorescence intensity on a unbleached/reference spot. $I_{Pre\ Bleached}$ and $I_{Pre\ Reference}$ were calculated by averaging the 10 measured fluorescence intensity values before the bleaching of the Bleached, and unbleached area, respectively. A non-linear least-square function was fitted to the normalized intensities from the recovery phase. The fit-function was:

$$f(t) = A(1 - \exp(-\lambda t)) + x_o \quad (6)$$

Where A and λ are fit parameters, and x_o correspond to the time point after bleaching, also referred as the start of the recovery phase. Then, by applying the protocol reported by Axelrod[16] and Soumpasis[17], the diffusion coefficient *D* of the lipids may be calculated via:

$$f = 0.32 \cdot \frac{r^2}{\tau_{1/2}} \quad (7)$$

Where τ is the half-recovery time, and *r* is the radius of the bleaching area.

Supplementary note 5: SUVs-to-GUV conversion efficiency calculation

The number of required lipid molecules to assemble a single giant unilamellar vesicle (GUV), $N_{Lip\ per\ GUV}$, can be written as:

$$N_{Lip\ per\ GUV} = 2 \cdot \frac{A_{SUV}}{A_{Head}} = 2 \cdot \frac{4\pi \cdot r_{GUV}{}^2}{A_{Head}} \tag{8}$$

Where A_{SUV} is the area of a GUV possessing a radius r_{GUV}, and A_{Head} is the area occupied by a single lipid head group.

The total number of lipids provided within the system, N_{Lip}, may be deduced from the total concentration of lipid used to produce dsGUVs:

$$N_{Lip} = c_{Lip} \cdot N_A \cdot V_{prod} \tag{9}$$

Where c_{Lip} is the lipid concentration encapsulated during dsGUV formation, N_A is the Avogadro number and V_{prod} is the total volume of aqueous phase used to generate the W/O emulsion.

Therefore, the theoretical number of GUVs possible to produce with the provided lipids can be described by:

$$N_{GUVs\ Theo} = \frac{N_{Lip}}{N_{Lip\ per\ GUV}} = \frac{c_{Lip} \cdot N_A \cdot V_{prod} \cdot A_{Head}}{8\pi \cdot r_{GUV}{}^2} \tag{10}$$

Similarly, the experimental number of GUVs generated and released into physiological condition can be approximated by evaluating the lipid contain, and hence number of free-standing GUVs, of the released aqueous phase by:

$$N_{GUVs\ Exp} = \frac{N_{Lip\ free-Standing\ GUVs}}{N_{Lip\ per\ GUV}} = f \cdot \frac{c_{GUV} \cdot V_{Well} \cdot N_A \cdot A_{Head}}{8\pi \cdot r_{GUV}{}^2} \tag{11}$$

Where f is a dilution factor associated to the dilution of the lipids, c_{GUV} is the lipid concentration associated to the free-standing GUVs measured by a calibration curve generated with the precursor SUVs, and V_{well} is the well's volume used to assess the fluorescence intensity by a plate reader measurement.

Then, the percentage of conversion efficiency, %Conversion, may be described as:

(12)

$$\%Conversion = 100\% \cdot \frac{N_{GUVs\ Exp}}{N_{GUVs\ Theo}} = 100\% \cdot f \cdot \frac{c_{GUV} \cdot V_{Well}}{c_{Lip} \cdot V_{prod}} \qquad (13)$$

With an average c_{GUV} of 7.75 µM, a well's volume of 100 µL, an initial lipid concentration c_{Lip} of 1.5 mM lipids and an aqueous volume of production V_{Prod} of 50 µL, the SUVs-toGUV conversion efficiency is roughly 20%.

Note* The factor *f* was evaluated as follow: Typically, 10 µL of free-standing GUVs were diluted to 100 µL (1:10 dilution), additionally, the initial 50 µL of lipid mixture used to assemble dsGUVs were released into a final 100 µL (thus, 1:2 dilution from initial lipid concentration). The resulting *f* factor corresponded to 20 in our experiment.

Video S1: pH sensitive SUVs entrapped in W/O droplets in presence of citrate buffer pH 5 using a two inlets microfluidics.

Video S2: Mechanical splitter module with a single inlet for production of multicompartment GUVs by microfluidics

Video S3: F-actin cytoskeleton inside a free-standing GUV

References

1. Heyes, J.; Palmer, L.; Bremner, K.; MacLachlan, I., Cationic lipid saturation influences intracellular delivery of encapsulated nucleic acids. *J Control Release* **2005,** *107* (2), 276-87.
2. Semple, S. C.; Akinc, A.; Chen, J.; Sandhu, A. P.; Mui, B. L.; Cho, C. K.; Sah, D. W.; Stebbing, D.; Crosley, E. J.; Yaworski, E.; Hafez, I. M.; Dorkin, J. R.; Qin, J.; Lam, K.; Rajeev, K. G.; Wong, K. F.; Jeffs, L. B.; Nechev, L.; Eisenhardt, M. L.; Jayaraman, M.; Kazem, M.; Maier, M. A.; Srinivasulu, M.; Weinstein, M. J.; Chen, Q.; Alvarez, R.; Barros, S. A.; De, S.; Klimuk, S. K.; Borland, T.; Kosovrasti, V.; Cantley, W. L.; Tam, Y. K.; Manoharan, M.; Ciufolini, M. A.; Tracy, M. A.; de Fougerolles, A.; MacLachlan, I.; Cullis, P. R.; Madden, T. D.; Hope, M. J., Rational design of cationic lipids for siRNA delivery. *Nat Biotechnol* **2010,** *28* (2), 172-6.
3. Whitehead, K. A.; Dorkin, J. R.; Vegas, A. J.; Chang, P. H.; Veiseh, O.; Matthews, J.; Fenton, O. S.; Zhang, Y.; Olejnik, K. T.; Yesilyurt, V.; Chen, D.; Barros, S.; Klebanov, B.; Novobrantseva, T.; Langer, R.; Anderson, D. G., Degradable lipid nanoparticles with predictable in vivo siRNA delivery activity. *Nat Commun* **2014,** *5* (1), 4277.
4. Liu, S.; Cheng, Q.; Wei, T.; Yu, X.; Johnson, L. T.; Farbiak, L.; Siegwart, D. J., Membrane-destabilizing ionizable phospholipids for organ-selective mRNA delivery and CRISPR-Cas gene editing. *Nat Mater* **2021,** *20* (5), 701-710.
5. Walsh, C. L.; Nguyen, J.; Tiffany, M. R.; Szoka, F. C., Synthesis, characterization, and evaluation of ionizable lysine-based lipids for siRNA delivery. *Bioconjug Chem* **2013,** *24* (1), 36-43.
6. Jayaraman, M.; Ansell, S. M.; Mui, B. L.; Tam, Y. K.; Chen, J.; Du, X.; Butler, D.; Eltepu, L.; Matsuda, S.; Narayanannair, J. K.; Rajeev, K. G.; Hafez, I. M.; Akinc, A.; Maier, M. A.; Tracy, M. A.; Cullis, P. R.; Madden, T. D.; Manoharan, M.; Hope, M. J., Maximizing the potency of siRNA lipid nanoparticles for hepatic gene silencing in vivo. *Angew Chem Int Ed Engl* **2012,** *51* (34), 8529-33.
7. Gruner, P.; Riechers, B.; Semin, B.; Lim, J.; Johnston, A.; Short, K.; Baret, J. C., Controlling molecular transport in minimal emulsions. *Nat Commun* **2016,** *7* (1), 10392.
8. Haller, B.; Gopfrich, K.; Schroter, M.; Janiesch, J. W.; Platzman, I.; Spatz, J. P., Charge-controlled microfluidic formation of lipid-based single- and multicompartment systems. *Lab Chip* **2018,** *18* (17), 2665-2674.
9. Holtze, C.; Rowat, A. C.; Agresti, J. J.; Hutchison, J. B.; Angile, F. E.; Schmitz, C. H.; Koster, S.; Duan, H.; Humphry, K. J.; Scanga, R. A.; Johnson, J. S.; Pisignano, D.; Weitz, D. A., Biocompatible surfactants for water-in-fluorocarbon emulsions. *Lab Chip* **2008,** *8* (10), 1632-9.
10. Baret, J. C., Surfactants in droplet-based microfluidics. *Lab Chip* **2012,** *12* (3), 422-33.
11. Skhiri, Y.; Gruner, P.; Semin, B.; Brosseau, Q.; Pekin, D.; Mazutis, L.; Goust, V.; Kleinschmidt, F.; El Harrak, A.; Hutchison, J. B.; Mayot, E.; Bartolo, J.-F.; Griffiths, A. D.; Taly, V.; Baret, J.-C., Dynamics of molecular transport by surfactants in emulsions. *Soft Matter* **2012,** *8* (41), 10618-10627.

12. Platzman, I.; Janiesch, J. W.; Spatz, J. P., Synthesis of nanostructured and biofunctionalized water-in-oil droplets as tools for homing T cells. *J Am Chem Soc* **2013,** *135* (9), 3339-42.

13. Scanga, R.; Chrastecka, L.; Mohammad, R.; Meadows, A.; Quan, P. L.; Brouzes, E., Click Chemistry Approaches to Expand the Repertoire of PEG-based Fluorinated Surfactants for Droplet Microfluidics. *RSC Adv* **2018,** *8* (23), 12960-12974.

14. Chowdhury, M. S.; Zheng, W.; Kumari, S.; Heyman, J.; Zhang, X.; Dey, P.; Weitz, D. A.; Haag, R., Dendronized fluorosurfactant for highly stable water-in-fluorinated oil emulsions with minimal inter-droplet transfer of small molecules. *Nat Commun* **2019,** *10* (1), 4546.

15. Weiss, M.; Frohnmayer, J. P.; Benk, L. T.; Haller, B.; Janiesch, J. W.; Heitkamp, T.; Borsch, M.; Lira, R. B.; Dimova, R.; Lipowsky, R.; Bodenschatz, E.; Baret, J. C.; Vidakovic-Koch, T.; Sundmacher, K.; Platzman, I.; Spatz, J. P., Sequential bottom-up assembly of mechanically stabilized synthetic cells by microfluidics. *Nat Mater* **2018,** *17* (1), 89-96.

16. Axelrod, D.; Koppel, D. E.; Schlessinger, J.; Elson, E.; Webb, W. W., Mobility measurement by analysis of fluorescence photobleaching recovery kinetics. *Biophys J* **1976,** *16* (9), 1055-69.

17. Soumpasis, D. M., Theoretical analysis of fluorescence photobleaching recovery experiments. *Biophys J* **1983,** *41* (1), 95-7.
